# Intrapulse multimodal four-wave sum mixing in the visible range from high contrast index grating with PMMA layer

**DOI:** 10.1038/s41377-025-02090-8

**Published:** 2026-01-05

**Authors:** Paolo Franceschini, Andrea Tognazzi, Evgenii Menshikov, Leonid Y. Beliaev, Radu Malureanu, Osamu Takayama, Ivano Alessandri, Alfonso Carmelo Cino, Domenico de Ceglia, Andrei V. Lavrinenko, Costantino De Angelis

**Affiliations:** 1 Department of Information Engineering, University of Brescia, Brescia, Italy; 2 National Institute of Optics-National Research Council, Brescia, Italy; 3 Department of Engineering, University of Palermo, Palermo, Italy; 4 School of Physics and Engineering, ITMO University, St. Petersburg, Russia; 5 Department of Electrical and Photonics Engineering, Technical University of Denmark, Kongens Lyngby, Denmark; 6 DTU Nanolab, National Centre for Nano Fabrication and Characterization, Kongens Lyngby, Denmark

**Keywords:** Nonlinear optics, Nanophotonics and plasmonics

## Abstract

Nonlinear metasurfaces have emerged as powerful platforms for enhancing and controlling light-matter interactions at the nanoscale, enabling versatile and compact design of devices for frequency conversion processes. In this work, we report on the experimental observation and theoretical analysis of intrapulse four-wave sum mixing (FWSM) in a high-index contrast grating (HCG) supporting quasi-bound states in the continuum (q-BIC). By engineering a one-dimensional silicon-based HCG with an additional poly(methyl methacrylate) (PMMA) cladding layer, we achieve the simultaneous excitation of a q-BIC and a guided-mode resonance (GMR), enabling nonlinear coupling between the two modes. Broadband femtosecond excitation reveals multiple distinct spectral peaks in the visible range, attributed to FWSM processes involving different combinations of q-BIC and GMR frequencies. Fourier microscopy measurements further confirm the redistribution of the generated nonlinear signals among diffraction orders. Our results demonstrate a new approach to tailoring nonlinear frequency mixing through metasurfaces, leveraging the interaction of multiple non-local resonances, thus opening new pathways for tunable frequency conversion, all-optical signal processing, and nonlinear photonic devices.

## Introduction

In the last two decades, the field of photonics has witnessed an increasing interest towards optical metasurfaces^1,2^, platforms composed of arrays of light scatterers. These structures represent a powerful tool to tailor the transmitted, reflected, and diffracted electro-magnetic fields by precise manipulation of phase, amplitude, and polarization of the incoming light^3,4^. Due to their numerous applications, such as beam steering^5^, focusing^6^, optical switching^7,8^, or in complex scattering formations such as holograms^9,10^, metasurfaces find application in a variety of fields spanning telecommunication, quantum optics, and biophotonics^11^. Metasurfaces composed of interacting elements garnered increasing attention after the recent demonstration of the independent tunability of their spectral and spatial selectivity^12^. The origin of this highly precise and selective wavefront manipulation is encoded in non-local (i.e., spatially extended) resonant modes arising from the engineered distribution of the sub-wavelength meta-atoms. In recent years, a new paradigm for confining the resonant optical modes has been proposed, and it is based on bound state in the continuum (BIC)^13^, i.e., spatially bounded eigenstates of the optical system characterized by infinitely high quality factors (Q-factor)^14^. In general, BICs are described as non-radiating resonant modes in an open system, meaning that for these states the coupling with the radiating channels propagating outside the system does not occur^13^. Within this class, a sub-category is given by symmetry-protected BICs for which the restricted out-coupling is due to their robustness against slight structural imperfection^13^. However, when the alteration of opto-geometrical parameters - resulting from material absorption, technological imperfections, or roughness^14^ - is large enough to break symmetry, BICs turn into resonant modes with high (yet finite) quality factors, known as quasi-BIC (q-BIC) modes. This allows obtaining an energy exchange with the external modes, leading to a strong near-field enhancement to further boost optical linear and nonlinear light-matter interaction. Within this framework, diffractive non-local metasurfaces are opening new opportunities, given their capability of leveraging symmetry-protected scattering from q-BICs^14,15^. Starting from design principles deeply rooted in symmetry considerations, diffractive non-local metasurfaces based on q-BIC are very promising for applications^16,17^ ranging from on-chip photonics and optical communication^18,19^ to biological sensing^20^ and quantum technologies^21^. Moreover, metasurfaces supporting q-BIC have been successfully employed for second- and third-harmonic generation (THG) and, also, for more complex nonlinear processes involving multiple frequencies, like four-wave mixing (FWM). This has been demonstrated by combining q-BIC with Mie resonances^22^ or mixing different q-BICs^23^, the latter based on a complex design process (for instance, two-dimensional periodicity^23,24^) necessary to achieve multiple q-BICs in a single metasurface. One of the simplest realizations of diffractive non-local metasurfaces is given by one-dimensional periodic high-contrast gratings (HCG)^12,25,26^, i.e., light-diffracting devices composed of high refractive index bars surrounded by a low-index background. Compared to conventional gratings, HCGs have secured increasing attention due to their enhanced ability to boost the optical field localization. The presence of strong electric field confinement resulting from suitably designed sharp spectral resonances and the absence of phase-matching^27^ requirements given by the sub-wavelength interaction lengths represent a natural way to enhance the nonlinear processes efficiency paired with improved control on the radiation properties^28,29^. In this framework, applications of stimulated and spontaneous parametric processes in metasurfaces have been reported, such as non-linear holography^10^, Terahertz generation^30^, or non-linear imaging^31^.

A widespread material for HCG featuring q-BIC modes in the near infrared (NIR)^14,32,33^ is silicon (Si), due to its high-refractive index, low-absorption losses, high nonlinear coefficient, low-cost, mature nano-fabrication processes and metal-oxide-semiconductor compatibility. Given these properties, Si-platforms supporting q-BIC or high-order Mie-resonant modes have been successfully employed for THG^34^ and FWM^23,24,35,36^. In general, this third-order nonlinear effect consists of the interaction of three input photons (at frequencies ${{\rm{\omega }}}_{1}$, ${\omega }_{2}$, and ${\omega }_{3}$) mixed together to generate a new (output) photon at a different frequency ${\omega }_{{FWM}}$. Moreover, it is a versatile nonlinear process that allows to tune the frequency of the nonlinear generated signal by modifying the frequency of the pump^35^. In the degenerate case, two input photons have the same frequency (*e.g*., ${{\rm{\omega }}}_{2}={{\rm{\omega }}}_{3}$), thus the set of four frequencies are ${{\rm{\omega }}}_{{\rm{FWM}}}=2{{\rm{\omega }}}_{1,2}\mp {{\rm{\omega }}}_{2,1}$, referred^37^ as *difference* and *sum* for “-” and “+”, respectively. While in the first case the output photon is generated at a frequency similar or close to the input ones^38,39^, in the second case, the output photon can be generated in a completely different spectral range compared to the input ones (for instance, starting from input photons in the infrared (IR) is possible to generate a photon in the visible (VIS)^40,41^). From the experimental point of view, the latter process is usually achieved with a two-beams configuration^22,23,35,41–43^, in which the two input frequencies come from two different light sources, in order to ensure the proper excitation of the modes involved in the nonlinear mixing process. However, tuning the delay between two pulses often requires the use of mechanical delay lines, an approach that may suffer from environmental instabilities arising from the non-collinear propagation of the pumps.

In this work, we experimentally and theoretically investigate a degenerate intra-pulse four-wave sum mixing (FWSM) process resulting from the interaction between a q-BIC and a guided-mode resonance (GMR)^44^ occurring in a one-dimensional periodic HCG, based on Si as the third-order nonlinear medium. First, we engineer a Si-based HCG supporting q-BIC at telecom wavelengths, and we introduce an additional cladding layer to obtain a guided mode resonance (GMR) at the desired frequency close to the q-BIC. Then, we analyze the optical properties of the fabricated metasurface in linear and nonlinear regimes. By focusing on the THG process involving only q-BIC, we experimentally demonstrate a five-orders-of-magnitude enhancement of the THG at the resonant wavelength compared to the unpatterned sample for both covered and uncovered HCG. Additionally, we elucidate the nonlinear coupling between q-BIC and GMR giving rise to FWSM processes upon illumination with a single broadband beam, where intrapulse frequency mixing (thus collinear propagation) allows us to overcome the limits of the non-collinear configuration approach and ensures the simultaneous arrival of the input photons. Finally, we experimentally and theoretically study third-harmonic light redistribution between the diffraction orders near the q-BIC. Our work introduces an additional degree of freedom involving nonlinear interaction between two nonlocal resonances for the design of nonlinear non-local metasurfaces, paving the way to the realization of flexible nanophotonics devices for nonlinear applications with reduced complexity.

## Results

**Fig. 1:**
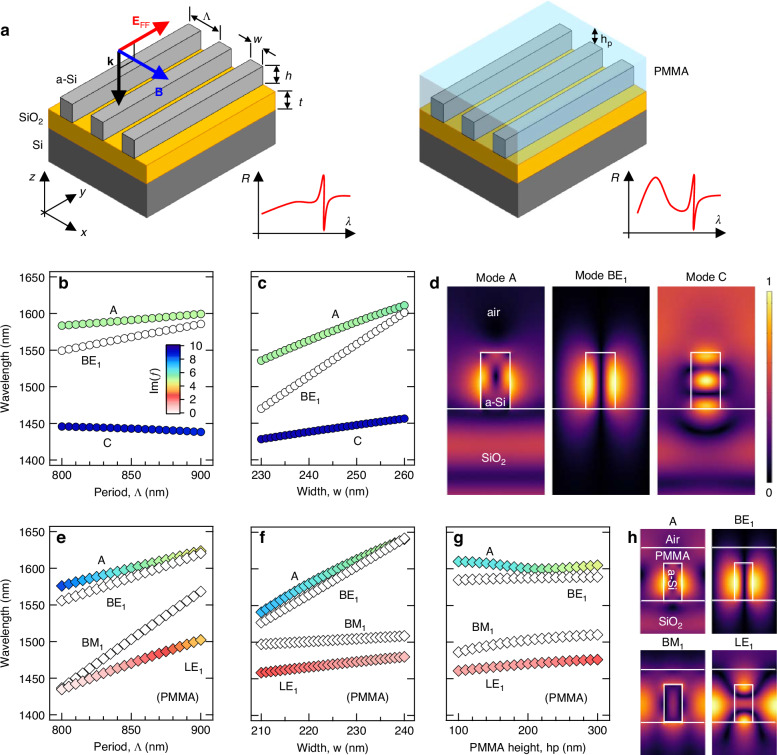
Metasurface Design. **a** Left: Schematic of the periodic grating (period $\varLambda$) consisting in amorphous silicon (a-Si) bars (height $h$ and width $w$) aligned along the $y$ direction and deposited on top of a silica (SiO_2_) buffer layer (thickness $t$). The underlying substrate is crystalline silicon (Si). Right: Schematic of the metasurface with additional PMMA layer (thickness ${h}_{p}$). The insets sketch the modulation of modes obtained when adding the PMMA layer. **b**, **c** Calculated dispersion curve of the modes eigenwavelength as a function of $\varLambda$ (**b**) and $w$ (**c**) without PMMA layer. **d** Spatial distribution in the ${xz}$ plane of the field amplitude (normalized absolute value) obtained from eigenmode analysis. **e**–**g** Calculated dispersion curve of the modes eigenwavelength as a function of $\varLambda$ (**e**), $w$ (**f**), and ${h}_{p}$ (**g**) with the PMMA layer. **h** Spatial distribution in the ${xz}$ plane of the field amplitude (normalized absolute value) obtained from eigenmode analysis. The colorscale in **b**, **c**, **e**–**g** represents the imaginary part of the eigenfrequency

The metasurface under analysis consists of a one-dimensional periodic amorphous-silicon (a-Si) HCG deposited on top of a silica (SiO_2_) buffer layer (thickness $t$ = 1140 nm) and supported by a Si substrate. The left panel of Fig. 1a schematizes the structure with period $\Lambda$ along the $x$ axis, the bars width $w$, and height $h$ = 489 nm. Here we underline that, given the selected geometry of the unit cell, the system may support modes with a different nature. Indeed, since one of the dimensions of the system extends to infinity, it can support BIC modes^13^. Moreover, the presence of the periodic repetition of low-loss a-Si bars on top of the SiO_2_ layer, which acts as a waveguide slab, makes the metasurface capable to support also *leaky* modes in the form of GMRs^45^. A poly(methyl methacrylate), PMMA, cladding layer (with thickness ${h}_{p}$ defined from the Si bars top) is introduced to tune the GMR spectral position (Fig. 1a, right panel). Indeed, PMMA is a well-known transparent polymer with stable properties, which can be easily deposited and removed^46^. Moreover, its response can be further tuned by using PMMA as a host medium for other polymers^47^. We engineer the metasurface to support resonant modes in the third operating optical communications window (1400-1600 nm) by a suitable optimization of the geometrical parameters $\Lambda$ and $w$. We also carefully consider the role of the periodicity of the designed metasurface when it interacts with light. Indeed, in the case of a grating, after a light beam excites the structure, the emitted radiation *might* propagate along directions (called radiation channels or diffraction orders) which differ from the impinging one^48^. However, a diffraction channel of order $m$ at wavelength $\lambda$ is opened (i.e., the radiation is emitted at an angle ${{\rm{\theta }}}_{m}$) in a medium with refractive index $n$ only if the condition ${{\rm{\theta }}}_{m}=\arcsin \left[m\cdot \lambda /\left(\Lambda \cdot n\right)\right]$ is fulfilled^14^. Therefore, we restricted the value of $\Lambda$ to be smaller than 900 nm in order to avoid the presence of diffraction orders for IR light (except the 0-th one), while allowing the coupling of high harmonics in the VIS to diffraction channels with $\left|m\right|\ge 1$.

To analyze the resonant wavelength and Q-factors of supported modes, we calculated the eigenmode spectrum of the metasurface for different values of the grating period, bar width, and PMMA thickness (see Supplementary Information for more details). The numerical calculations were performed by using COMSOL Multiphysics. Similarly to ref. ^33^, our analysis reveals that, without the PMMA layer, the structure supports three modes in the 1400–1600-nm spectral range, as suggested by the dispersion curves in Fig. 1b and c, where the color scale represents the losses (imaginary part of the eigenfrequency $f$). In particular, as shown by the modes spatial distribution in Fig. 1d (see also Sec. III.A of the Supplementary Information for more details), the metasurface features an electric/magnetic leaky mode at 1450 nm (mode C), an electric bounded mode at 1550 nm (mode BE_1_), and an electric/magnetic leaky mode at 1600 nm (mode A).

As expected, the mode eigenwavelength (real part of the eigenfrequency $f$) slowly varies with the period (Fig. 1b), while the rate strongly increases when the width dependence is considered (Fig. 1c). Indeed, in the case of BE_1_, a variation of 10% in $w$ corresponds to a spectral shift of nearly 150 nm, highlighting the demand for extremely high lateral resolution to tailor the metasurface properties. The eigenmode analysis reveals a strong field localization occurring at the a-Si bars sidewalls (modes A and BE_1_) or inside the volume (mode C). Then, given its properties, we consider the role of the PMMA layer surrounding the a-Si bars on the mode dispersion (Fig. 1e–h). As can be seen in Fig. 1e and f, the presence of the PMMA layer partially modifies the properties and the number of modes supported by the metasurface in the 1400-1600 nm spectral range.

Indeed, the PMMA-based metasurface features four modes: an electric leaky mode (LE_1_) at ~1450 nm, a magnetic bounded mode (BM_1_) at ~1500 nm, an electric bounded mode (BE_1_) at ~1580 nm, and an electric/magnetic leaky mode (A) at ~1600 nm. At this point it is worthy to underline that, given the antisymmetric nature of the spatial distribution with respect to the ${yz}$ plane (which prevents any coupling to a symmetric plane wave) and the zero-valued imaginary part of its eigen-frequency (confirming the absence of any radiating channel propagating outside the system), the mode BE_1_ consists in a symmetry-protected BIC mode^13,49,50^. This is further corroborated by the expected^51,52^ quadratically decaying nature of the $Q$-versus-$k$ scaling law for small values of ${K}_{x}$ ($Q\propto 1/{K}_{x}^{2}$, with ${K}_{x}$ being the in-plane component of the Bloch wave vector) shown by the BE_1_ mode (see Supplementary Fig. S7 for more details). On the other hand, the symmetric distribution of the spatial profile featuring a not-fully confined field with a radiative component along the vertical direction allows to conclude that the LE_1_ mode is a GMR^45,50^. Finally, Fig. 1g suggests that the PMMA thickness (${h}_{p}$) does not alter significantly the modes dispersion. Interestingly, the eigenmode analysis reveals that, compared to the others, mode BM_1_ is localized within the PMMA layer in the region between Si bars, thus explaining the strong dependence of the corresponding eigenwavelength on $\Lambda$, as shown by mode spatial distribution in Fig. 1h. Therefore, in order to obtain a metasurface featuring BE_1_ resonance in the third-operating telecom window, we fabricated a first sample with the following values of the geometrical parameters: $\Lambda$ = 840 nm, $w$ = 225 nm, and ${h}_{p}$ = 200 nm. As a reference, we then fabricated a sample (without PMMA layer, which will be labeled as REF in the following) with $\Lambda$ = 840 nm and $w$ = 255 nm, the latter being chosen in order to obtain the similar spectral position for BE_1_ (~1580 nm).

**Fig. 2:**
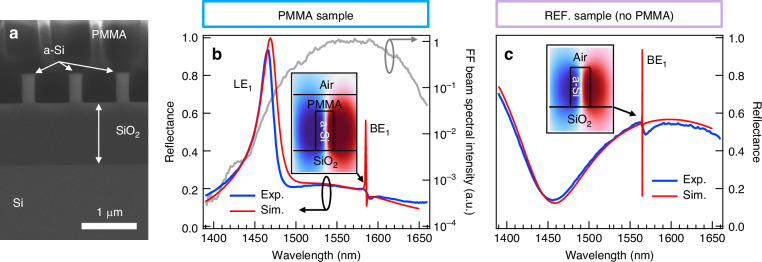
Linear Characterization. **a** SEM image of the fabricated metasurface with additional PMMA layer (*h*_*p*_ = 2 μm thickness). **b**, **c** Measured and calculated reflectance spectra of the photonic metasurface (**b**, PMMA: sample with PMMA layer) with and (**c**, REF: reference sample) without PMMA layer obtained for TE probe light (experiment: blue solid line, simulation: red solid line). The insets display the calculated spatial distribution of the out-of-plane component of the electric field, ${E}_{y}$(negative and positive values are displayed in blue and red colors, respectively), for BE_1_ mode in the ${xz}$ plane (obtained from plane-wave-illumination-based numerical simulations). The gray solid line in **b** (right axis) denotes the spectrum of the fundamental frequency excitation beam adopted in the broadband regime measurements

To probe that the fabricated samples (see Scanning Electron Microscope, SEM, image in Fig. 2a) support the BE_1_ modes, we measured linear reflectance spectra by using a home-made spectrometer^8^. In particular, since the probe light impinges at normal incidence on the sample surface, a thin-lens has been introduced to access the symmetry-protected BIC (see Materials and Methods for more details). Figure 2b, c display the experimental reflectance spectrum (blue solid line) of PMMA and REF samples (panel b and c, respectively) obtained for linear polarization parallel to the direction of the grating bars ($x$ axis, transverse-electric, TE). Both spectra are characterized by broadband and shallow background peaks ascribed to Fabry-Pérot resonances due to the multilayer nature of the metasurface. A sharp peak appears for both samples in the 1560-1590 nm wavelength range, which is the signature of the designed bounded mode BE_1_ and manifests itself as a q-BIC resonant mode (close to the BIC state) in the form of a Fano resonance in the optical response spectrum. For the PMMA sample also the spectral signature of the GMR (mode LE_1_) is visible. Linear spectrum for TM (transverse magnetic) polarization shows the BM_1_ signature feature (see Supplementary Fig. S2 for more details). By using input values of the geometrical parameters extracted from the SEM images and optical properties obtained from ellipsometry measurements, we performed numerical simulations to investigate the field distribution in the fabricated metasurfaces (see Materials and Methods for more details). In particular, to improve the matching between the theoretical profiles (solid red curves in Fig. 2b, c) and the experimental data, we applied an optimization procedure to the sum of objective functions ${R}_{d}={\sum }_{i}{\left[{R}_{d}^{\exp }\left({\lambda }_{i}\right)-{R}_{d}^{{sim}}\left({\lambda }_{i}\right)\right]}^{2}$ ($d$=TE, TM) based on the Nelder-Mead method, where the values of the geometrical parameters act as free parameters. We underline that the optimization procedure has been performed simultaneously on both linear reflectance spectra measured with the TE and TM-probe light. The analysis provides $\Lambda$ = 850 nm, $w$=226 nm, and ${h}_{p}$ = 203 nm in the case of the sample with PMMA layer, while, for the REF sample, $\Lambda$ = 850 nm and $w$=248 nm. The value of the geometrical parameters obtained from numerical analysis matches (within the experimental error bar ascribed to the refractive index dispersion and SEM resolution) with the input one. Deviations between the values employed as input for the fabrication processes and those retrieved from the analysis are consistent with the fabrication tolerances. The discrepancy regarding the bandwidth of the BE_1_ mode between the experimental and numerical profiles can be ascribed mainly to three aspects: (i) the finite spectral resolution of the spectrometer adopted (1.5 nm at 1500 nm), (ii) the angular bandwidth of the radiation impinging onto the metasurface (i.e., due to the numerical aperture of the lens of 0.09, the angle of incidence of the components within the probe light ranges from 0° to +5°), and (iii) the size-effect due to the illumination of a finite number of unit cells.

**Fig. 3:**
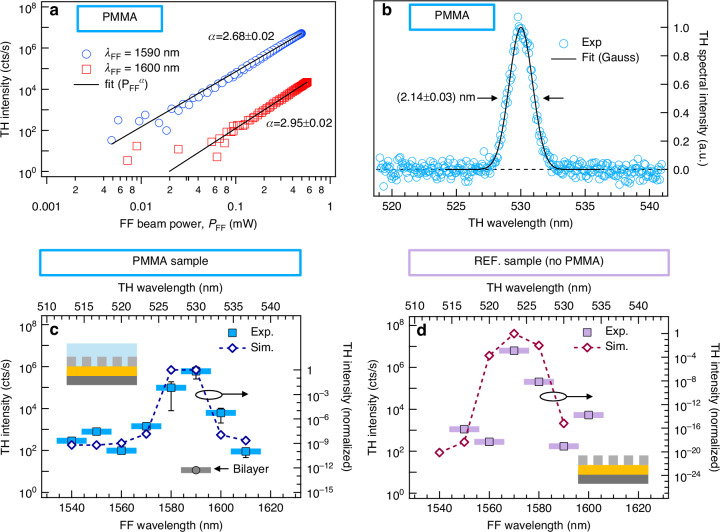
Third-Harmonic Generation - Narrowband Excitation Regime. **a** Excitation-dependent third-harmonic (TH) signal intensity upon illumination with narrowband ($\varDelta {\lambda }_{{FF}} \sim$ 12 nm) beam centered at ${\lambda }_{{FF}}$. The solid black line is the fit of a power-law profile to the experimental data (markers). The value of the exponent $\alpha$ retrieved from the analysis is indicated near the corresponding data. **b** Spectrum of the TH radiation obtained upon illuminating the PMMA metasurface with a narrowband pump beam TE polarized with ${\lambda }_{{FF}}$ = 1590 nm and $\varDelta {\lambda }_{{FF}}=\left(11.6\pm 0.1\right)$ nm. A Gaussian profile (black solid line) has been fitted to the data (markers). The retrieved value of the TH spectral bandwidth is $\varDelta {\lambda }_{{TH}}=\left(2.14\pm 0.03\right)$ nm. **c**, **d** Measured (square markers, left axis) and calculated (markers and dotted line, right axis) TH intensity as a function of ${\lambda }_{{FF}}$ (at fixed ${P}_{{FF}}$ = 250 μW) for the metasurface (**c**) with and (**d**) without PMMA layer. The thick horizontal colored bar highlights the 12-nm spectral bandwidth of the FF excitation adopted in the experiment. The circular marker in panel **c** denotes the TH intensity measured from the unpatterned multilayer structure

Given the strong field enhancement occurring at the BE_1_ resonant frequency, we experimentally investigated the nonlinear signal generation following illumination by an ultrafast laser pulse with fundamental frequency (FF) tuned to spectrally match the q-BIC resonance. Moreover, due to the collective nature of the BE_1_ mode, the FF beam is required to excite a proper number of unit cells in order to observe the effects of the strong field confinement. We employed a nonlinear microscopy setup working in reflection geometry, similar to the one in^53^, to excite the metasurface with a collimated (plane wave) FF beam (beam waist ${w}_{0}$ = 35 μm, corresponding to the illumination of ~10^2^ unit cells) and collect the backward emitted nonlinear radiation with a large numerical aperture (NA) objective (see Methods for more details). We performed nonlinear generation measurements in the *narrowband* regime by using IR FF excitations with spectral bandwidth (full width at half maximum) of $\varDelta {\lambda }_{{FF}}$ = 12 nm. Figure 3a shows power-dependent measurements (markers) of the nonlinear signal intensity generated by the metasurface for non-resonant (${\lambda }_{{FF}}$ = 1600 nm) and resonant (${\lambda }_{{FF}}$ = 1590 nm) pump wavelength values (red and blue markers, respectively) near the BE_1_ resonance frequency (${P}_{{FF}}$ denotes the average power of the incident pulsed FF beam measured at 500 kHz repetition rate). Here, the direction of the linear polarization of the FF beam is parallel to the grating bars (a detailed polarization-resolved study of the nonlinear signal is reported in the Supplementary Information Fig. S3). A power-law profile ($\propto {P}_{{FF}}^{\alpha }$, black solid line) has been fitted to the experimental data at ${\lambda }_{{FF}}$ = 1600 nm and the analysis provides ${\rm{\alpha }}=\left(2.95\pm 0.02\right)$, which suggests a third-order behavior of the process (see Section IB of the Supplementary Information for more details). The TH nature of the nonlinear interaction under analysis is confirmed by the spectrum (see Fig. 3b) of the radiation emitted upon illumination with TE-polarized FF beam at ${\lambda }_{{FF}}$ = 1590 nm. Indeed, both the spectral position ${\lambda }_{{TH}}=\left(530.01\pm 0.01\right)$ nm and the bandwidth amplitude $\varDelta {\lambda }_{{TH}}=\left(2.14\pm 0.03\right)$ nm are consistent with the FF beam spectral properties (see Supplementary Information Sec. II.D for more details). When the FF beam polarization changes from TE to TM, the TH signal intensity decreases by nearly two orders of magnitude as a result of the polarization selectivity of mode BE_1_^33^ (see Fig. S3 of the Supplementary Information). We underline that, from an experimental point of view, each data point (i.e., each marker) in Fig. 3a represents the output of the single-photon avalanche detector, which provides the total power of the TH radiation generated by the narrow band excitation at the FF. This represents a spectrally integrated value over the spectral interval determined by the size of the bandwidth of the narrow-band excitation.

The measured TH signal intensity as a function of ${\lambda }_{{FF}}$ at fixed ${P}_{{FF}}$ value for the PMMA sample is displayed in Fig. 3c (markers). This profile demonstrates that at resonant excitation of the BE_1_ (i.e., for ${\lambda }_{{FF}}$ = 1590 nm), the TH signal intensity increases up to three orders of magnitude compared to the non-resonant excitation and up to five orders of magnitude compared to the corresponding unpatterned platform (i.e., a bilayer thin-film structure deposited on Si substrate with the same thickness values for the upper Si layer and for the buffer SiO_2_ layer). To gain further insights on the nonlinear generation process occurring in the metasurface, we performed numerical simulations of the THG process. In the numerical model, we assume that the THG process occurs within the grating bars. Moreover, since the mode BE_1_ under analysis has a spectral bandwidth ${\rm{\gamma }}$ much narrower than the FF beam bandwidth $\varDelta {\lambda }_{{FF}}$, each point of the simulated TH signal has been calculated taking into account the contributions provided by various spectral components within the bandwidth $\varDelta {\lambda }_{{FF}}$ (see Supplementary Information Sec. IV.A for more details). Since the spectral tuning of ${\lambda }_{{FF}}$ (with a finite bandwidth) selects the excitation interval, the numerical analysis confirms that the enhancement of the TH signal intensity is ascribed to the strong field confinement occurring at BE_1_ (see Fig. 3c). As shown in Fig. 3d, the experimental and numerical analysis performed on the REF sample reveals that the presence of the covering PMMA layer does not significantly decrease the efficiency of the THG process. Overall, the experimental data in Fig. 3c and d allow to estimate the third-harmonic generation efficiency ${\eta }_{{TH}}$. Following refs. ^54^, we calculated the THG efficiency as ${\eta }_{{TH}}={I}_{{TH}}\cdot {E}_{{ph},{TH}}/{P}_{{FF}}$, where ${I}_{{TH}}$ and ${E}_{{ph},{TH}}$ are the photon count-rate (expressed in counts/s) and the photon energy of the TH radiation, respectively. For the resonant excitation, we obtain ${\eta }_{{TH}}=9.0\cdot {10}^{-10}$ and ${{{\eta }}}_{{{TH}}}=9.5\cdot {10}^{-9}$ for the sample with and without PMMA layer, respectively (see Supplementary Information Sec. II.E for more details).

**Fig. 4:**
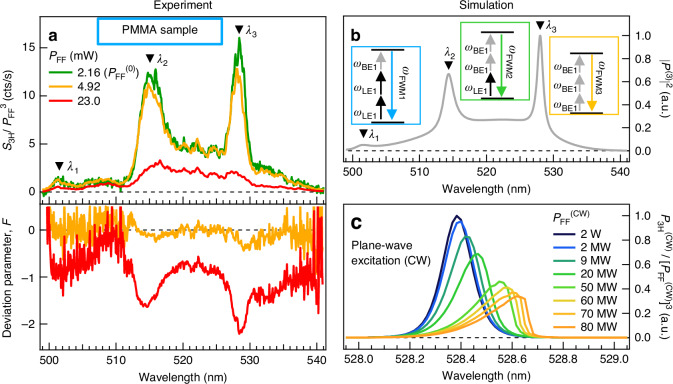
Four-Wave Sum Mixing Process - Broadband Excitation Regime. **a** Top panel: Measured spectrum of the nonlinear radiation obtained by broadband pump illumination of the PMMA metasurface for three excitation power levels, ${P}_{{FF}}$. The labels identify the peaks ascribed to four-wave mixing processes. Each spectrum is normalized to the third power of its fundamental frequency beam power. Bottom panel: deviation parameter $F$ calculated according to Eq. (1) with ${P}_{{FF}}^{\left(0\right)}$ = 2.16 mW. **b** Intensity of the third-order polarization ${\left|{P}^{\left(3\right)}\right|}^{2}$ calculated according to Eq. (2). The insets show the diagrams of the degenerate four-wave sum mixing processes under analysis. **c** Excitation-dependent third harmonic generation efficiency ${\xi }_{3H}={P}_{3H}/{P}_{{FF}}^{3}$ calculated within plane-wave excitation condition in the spectral region near the BE_1_ mode

As shown in Fig. 3a, with the BE_1_ resonant excitation, at high-level pump values, the TH signal shows a weak saturation effect, suggesting a deviation from the cubic behavior; this is also confirmed by the analysis, which provides ${\rm{\alpha }}=\left(2.68\pm 0.02\right)$ as the exponent of the theoretical power-law profile at ${\lambda }_{{FF}}$ = 1590 nm. In order to better investigate the origin of the deviation from the cubic behavior, we performed wavelength-resolved excitation-dependent measurements of the nonlinear signal generated after excitation by a broadband ($\varDelta {\lambda }_{{FF}}=\left(97\pm 1\right)$ nm) beam at the FF, whose spectrum is shown in Fig. 2b (gray curve, right axis). The top panel of Fig. 4a displays the spectra of the generated nonlinear radiation in the VIS range (${S}_{3H}$) obtained from the PMMA sample, where each curve is normalized to the third power of the FF beam intensity (${P}_{{FF}}^{3}$). The displayed spectra reveal a rich nonlinear physics involving several modes. We recognize the presence of three spectral peaks (${\lambda }_{1}$ at 501 nm, ${\lambda }_{2}$ at 515 nm, and ${\lambda }_{3}$ at 528 nm) and the strong deviation from the cubic regime (sub-cubic in our case) for the nonlinear process. The latter aspect can be conveniently visualized (bottom panel in Fig. 4a) by introducing the deviation factor^55^:

1 $$F={\text{ln}}\left[\frac{{S}_{3H}\left({P}_{FF}\right)}{{S}_{3H}\left({P}_{FF}^{\left(0\right)}\right)\cdot {\left({P}_{FF}/{P}_{FF}^{\left(0\right)}\right)}^{3}}\right]$$

where ln denotes the natural logarithm, ${S}_{3H}\left({P}_{{FF}}\right)$ is the spectrum of the third-order nonlinear radiation generated by the FF excitation with power ${P}_{{FF}}$, and ${P}_{{FF}}^{\left(0\right)}$ is the minimum pump power value (of the broadband pump pulse) used in this measurement (${P}_{{FF}}^{\left(0\right)}$ = 2.16 mW). Within this framework, $F=0$ denotes a cubic power dependence, while negative values ($F < 0$) correspond to sub-cubic power dependence. The presence of three peaks in the spectrum can be ascribed to intrapulse FWSM resulting from the large bandwidth of the fundamental beam exciting the metasurface. Indeed, as shown in Fig. 2b, the spectrum of the FF broadband beam overlaps not only with the sharp BE_1_ at ${\lambda }_{{\rm{BE}}1}\propto {{\rm{\omega }}}_{{\rm{BE}}1}^{-1}$, but also with the LE_1_ mode with ${\lambda }_{{\rm{LE}}1}\propto {{\rm{\omega }}}_{{\rm{LE}}1}^{-1}$ (with ${{\rm{\omega }}}_{{\rm{BE}}1}$ and ${{\rm{\omega }}}_{{\rm{LE}}1}$ being the angular/optical frequencies). Therefore, the three peaks ${\lambda }_{1}$, ${\lambda }_{2}$, and ${\lambda }_{3}$ in Fig. 4a can be described as the result of the FWSM processes of the form ${{\rm{\omega }}}_{{\rm{FWM}}1}=2{{\rm{\omega }}}_{{\rm{LE}}1}+{{\rm{\omega }}}_{{\rm{BE}}1}$, ${{\rm{\omega }}}_{{\rm{FWM}}2}={{\rm{\omega }}}_{{\rm{LE}}1}+2{{\rm{\omega }}}_{{\rm{BE}}1}$, and ${{\rm{\omega }}}_{{\rm{FWM}}3}=3{{\rm{\omega }}}_{{\rm{BE}}1}$, respectively (see diagrams in Fig. 4b). To further support our description, we calculate the third-order polarization in the time domain (following Ref. ^56^) as

2 $${P}_{{eff}}^{\left(3\right)}\left(t\right)\propto {\left[{{\mathcal{F}}}^{-1}\left\{{t}_{{eff}}\left({\rm{\omega }}\right)\cdot {E}_{{FF}}\left({\rm{\omega }}\right)\right\}\left(t\right)\right]}^{3}$$

where ${{\mathcal{F}}}^{-1}$ denotes the inverse Fourier-Transform operation, ${t}_{{eff}}\left(\omega \right)$ is an effective linear response function of the metasurface taking into account the presence of multiple resonances (whose individual spectral profile is described as Fano lineshape^57^, see Supplementary Information Sec. IV.C for more details), and ${E}_{{FF}}\left(\omega \right)$ is the spectral profile of the electric field at FF (retrieved from Fig. 2b). Figure 4b shows the third-order polarization at the TH frequency ${\left|{P}^{\left(3\right)}\left({\rm{\omega }}\right)\right|}^{2}={\left|{\mathcal{F}}\left\{{P}_{{eff}}^{\left(3\right)}\left(t\right)\right\}\left({\rm{\omega }}\right)\right|}^{2}$. Although being a simplified approach, our model is capable to correctly grasp the main spectral features, thus confirming the nonlinear frequency mixing of the fundamental pump beam spectral components mediated by the LE_1_ and BE_1_ modes. The $F$ parameter in Fig. 4a highlights that, at low ${P}_{{FF}}$ values (yellow solid line), the sub-cubic deviation only affects the spectral properties in the neighboring region between peaks ${\lambda }_{2}$ and ${\lambda }_{3}$. However, at larger pump power values (red solid line), the deviation becomes more pronounced. To investigate the power-induced modifications of the third-order nonlinear spectrum, we perform single-frequency numerical simulations of the nonlinear process in the spectral region of the BE_1_ resonance. In particular, we simulate the TH generation in the presence of the two-photon absorption and Kerr effect. These additional mechanisms have been taken into account by adopting a complex third-order susceptibility at FF: ${{\rm{\chi }}}^{\left(3\right)}\left({\rm{\omega }}\right)=\left(7+{\rm{j}}0.8\right)\times {10}^{-20}$ m^2^/V^2^ and ${{\rm{\chi }}}^{\left(3\right)}\left(3{\rm{\omega }}\right)=\left(5+{\rm{j}}3\right)\times {10}^{-19}$ m^2^/V^2^. These values, taken from Ref. ^58^ have been calculated by using a Duffing-oscillator model for the polarization, which provides a method to evaluate the spectral properties of the third-order susceptibility of a material starting from its first-order susceptibility. As shown in Fig. 4c, when the FF beam power increases (with ${P}_{{FF}}^{\left({CW}\right)}$ being the power of a monochromatic continuous plane-wave excitation), the spectral resolved TH efficiency, ${\xi }_{{TH}}\left(\lambda \right)={P}_{{TH}}\left(\lambda \right)/{P}_{{FF}}^{3}\left(\lambda \right)$, decreases due to the two-photon absorption effect, and the Kerr-type nonlinearity induces a spectral shift of the TH peak.

Given the geometrical properties of the designed metasurface, since the generated TH lies in the VIS spectral range, its propagation (forward and backward with respect to the metasurface) occurs not only through the diffraction channel of order $m=0$, but also through higher-order ones. Therefore, we employ the Fourier Microscopy technique to measure the intensity and angular distribution of the diffraction orders of the TH radiation. We access the in-plane momentum space ($k$-space) by imaging the back focal plane (BFP) of the nonlinear microscope detection objective on a detector^59^ (see Supplementary Information for more details).

**Fig. 5:**
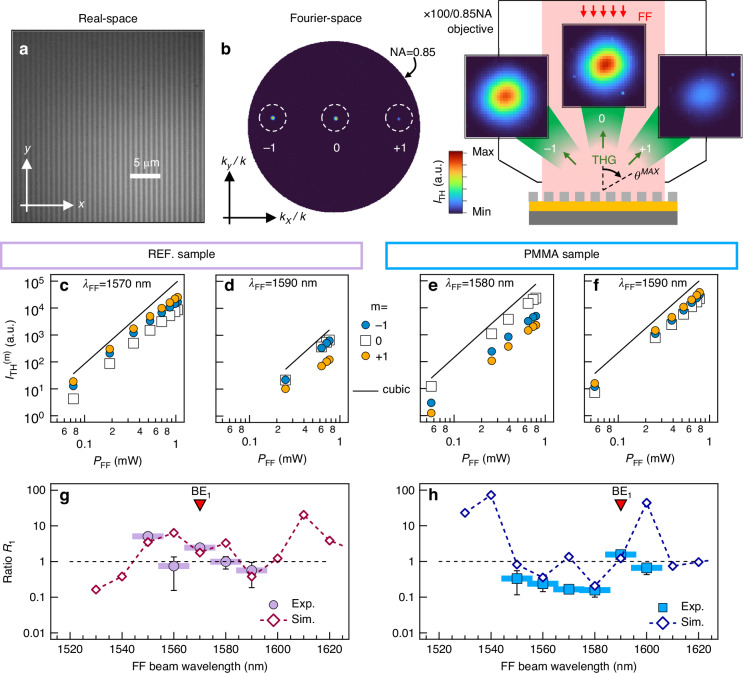
Fourier Microscopy. **a** Real-space image (top view) of the REF sample. **b** Left: Fourier-space image of the TH radiation ${I}_{{TH}}\left({k}_{x},{k}_{y}\right)$ emitted by the REF sample upon illumination with a narrowband ($\varDelta {\lambda }_{{FF}}=\left(11.6\pm 0.1\right)$ nm) FF beam at ${\lambda }_{{FF}}$ = 1590 nm. The outer circumference represents the boundary of the experimentally accessible $k$-space (NA = 0.85, ${\theta }^{{MAX}}=\arcsin \left(0.85\right)\,\simeq$58°). The dashed white circles indicate the diffraction orders (DOs) at the TH. Right: sketch of the FF illumination and collection of the back-emitted diffraction TH orders with the corresponding detailed view (near the DOs peak) of the BFP map on the left. **c**–**f** Measured excitation-dependent intensity of the TH radiation emitted through the $m$ th diffraction order ${I}_{{TH}}^{\left(m\right)}$. The black solid line serves as a visual guide, indicating the theoretical cubic dependence of a THG process. **g**, **h** Ratio ${R}_{1}=\left({I}_{{TH}}^{\left(-1\right)}+{I}_{{TH}}^{\left(+1\right)}\right)/\left(2{I}_{{TH}}^{\left(0\right)}\right)$ as a function of ${\lambda }_{{FF}}$ obtained from experimental data (markers) and simulations (markers and dotted line) for REF (**g**) and PMMA (**h**) sample. The red triangle denotes the spectral position of BE_1_ mode

For illustrative purposes, Fig. 5a and b show the real- and Fourier-space images of the REF sample, respectively (similar images are obtained for the PMMA sample). In particular, Fig. 5a displays the top view of the grating metasurface with the bars aligned along the $y$-axis (vertical direction). When the narrowband ($\varDelta {\lambda }_{{FF}}$ = 12 nm) TE FF beam excites the grating, the intensity distribution in the Fourier-space of the back-emitted TH radiation, ${I}_{{TH}}\left({k}_{x},{k}_{y}\right)$, manifests itself as in the BFP map shown in Fig. 5b (obtained at ${\lambda }_{{FF}}$ = 1590 nm). Each of the three peaks appearing along the horizontal direction represents the in-plane components (in the ${k}_{x}{k}_{y}$ plane) of the wave vector corresponding to a specific diffraction order of the TH radiation: $m=-1$, $m=0$, and $m=+1$ (whose detailed view is shown in the left, central, and right panel of the sketch in Fig. 5b). At this stage it is important to underline two aspects. First, given the experimental numerical aperture (NA = 0.85) of the employed collection objective, any diffraction channel emitted at an angle larger than ${{\rm{\theta }}}^{{\rm{MAX}}}=\arcsin \left(0.85\right)\simeq$ 58° falls outside the detection region. Second, in the case of the sample without the PMMA layer, only (backward) diffraction orders $-1$, $0$, and $+1$ are allowed (i.e., those displayed in Fig. 5b). In the case of the sample with the PMMA layer, (backward) orders $\pm 2$ exist in the PMMA layer but cannot propagate in air due to total internal reflection at the PMMA/air interface. Thus, also for the PMMA samples only 0,$\pm 1$ orders are allowed to back propagate in air.

The analysis of the peaks position in Fig. 5b provides the value of the emission angle ${{{\theta }}}_{1}=\arcsin \left({{k}}_{{\rm{x}}}/{k}\right)$ = (38° 40’ ± 0° 20’) for $m=\left|1\right|$, thus, a value of $\Lambda =\left(848\pm 5\right)$ nm for the grating period, which is consistent with those obtained from the numerical simulations of the reflectance spectra (Fig. 2b) and from the optical microscope image in Fig. 5a, the latter being $\left(846\pm 3\right)$ nm. In order to gain further insights on the fraction of TH radiation carried by each diffraction order (i.e., how the intensity of the back-emitted TH radiation is distributed over the various radiation channels), we performed excitation-dependent measurements of the TH intensity of the three diffraction orders in the *narrowband* regime ($\varDelta {\lambda }_{{FF}}$ = 12 nm) at different pump wavelength ${\lambda }_{{FF}}$. Figure 5c–f show the intensity of the TH radiation emitted in the $m$-th diffraction channel (${I}_{{TH}}^{\left(m\right)}$) as a function of the incident power of the narrowband FF beam in the case of the resonant and non-resonant excitation of BE_1_, for both REF and PMMA samples. The intensity value ${I}_{{TH}}^{\left(m\right)}$ was calculated by integrating the TH signal amplitude over the region ${\Omega }_{{\rm{m}}}$ in the $k$-space where the diffraction order appears (white dashed circles in Fig. 5b) and then, normalized to the area of the region ${\Omega }_{{\rm{m}}}$ itself, i.e., ${I}_{{TH}}^{\left(m\right)}={\int }_{{\Omega }_{{\rm{m}}}}{I}_{{TH}}\left({k}_{x},{k}_{y}\right)\text{d}{k}_{x}\text{d}{k}_{y}/{\int }_{{\Omega }_{{\rm{m}}}}\text{d}{k}_{x}\text{d}{k}_{y}$ (given the symmetry of the metasurface and the polarization of the FF beam, any difference in the amplitude of the signal ascribed to $-1$ and $+1$ orders is attributed to slight misalignment between the beams and the optical elements). The TH intensity profiles measured (markers) for resonant excitation condition (Fig. 5c and f) reveal that the above-mentioned deviation from the cubic dependence (black solid line) affects all three diffraction orders. Interestingly, the data displayed in Fig. 5c–f reveal that, given the total intensity of the TH radiation back-emitted by the metasurface, the fraction of TH signal carried by order 0 increases or decreases with respect to that carried by order $m=\left|1\right|$ when the wavelength ${\lambda }_{{FF}}$ changes. To better visualize the TH radiation redistribution among the diffraction orders, in Fig. 5g and h (REF and PMMA sample, respectively) we report the FF-wavelength-dependent average ratio ${R}_{1}=\left({I}_{{TH}}^{\left(-1\right)}+{I}_{{TH}}^{\left(+1\right)}\right)/\left(2{I}_{{TH}}^{\left(0\right)}\right)$ (markers) calculated from the experimental data in Fig. 5c–f (see Supplementary Information Fig. S4 for additional data). For both samples, the value of the ratio changes as a function of ${\lambda }_{{FF}}$. Similarly to the analysis of the results in Fig. 3c and d, we estimated the ratio ${R}_{1}$ starting from numerical simulations. The contribution due to the various spectral components within the experimental FF beam to the intensity of the three diffraction orders have been evaluated by taking into account the spectral envelope of the FF beam itself (see Supplementary Information Section IV.D for more details). As shown in panels Fig. 5g and h, the calculated intensity ratio (marker and dashed line) allows to reproduce quite well the experimental data by assuming off-normal incidence angles of the FF excitation of 5° and 2° (for the REF and PMMA sample, respectively), which are consistent with those retrieved from the analysis of the BFP images. The discrepancy observed for some value of ${\lambda }_{{FF}}$ can be ascribed mainly to (i) a slightly different value of the incident angle of the FF excitation beam and to (ii) the *effective* nature of the method employed to take into account the finite bandwidth of the FF beam (adopted in the experiment) in the evaluation of the ratio ${R}_{1}$ from the numerical simulations (which are based on a monochromatic excitation at FF). Briefly, our analysis suggests that the TH radiation re-distribution observed in the BFP images (i.e., a measurement of the far-field properties of the radiation) is the result of the modulation of the near-field distribution occurring in the spectral range near the BE_1_. In addition, the presence of the PMMA layer introduces a further modulation due to the Fabry-Pérot effects.

## Discussion

Nonlinear metasurfaces^60–62^ represent a powerful tool in the field of nonlinear generation since, unlike other nonlinear platforms, such as nonlinear crystals, fibers, and waveguides, their nonlinear efficiency does not suffer from propagation losses and phase matching terms^56^. Indeed, their promising nonlinear generation performances are based on a strong light-matter interaction resulting from the strong field enhancement induced by resonances. Here, we proposed a Si-based diffractive nonlocal metasurface consisting in a one-dimensional periodic HCG featuring optical modes in the NIR, and we investigated its third-order generation properties upon narrowband and broadband excitation conditions. Our results demonstrate THG and intrapulse sum-FWM in nonlocal metasurfaces by exploiting a q-BIC resonant mode and a GMR. The good agreement between numerical simulations and the linear spectroscopy measurements demonstrates the high quality of the fabricated metasurfaces. The analysis of TH radiation emitted upon illumination with a narrowband FF excitation in the q-BIC spectral range reveals that the presence of the PMMA preserves the efficiency of THG, thus indicating that PMMA is a promising constituent for the development of nonlinear devices. Moreover, the study of nonlinear radiation emitted from broadband excitation discloses also the possibility to exploit four-wave mixing processes involving multiple resonances, thus suggesting the promising role of the cladding layer as an additional degree of freedom in tuning the spectral performance of optical metasurfaces (*e.g*., with potential application in CARS or Raman scattering amplification). Finally, Fourier non-linear microscopy measurements revealed the additional capability of PMMA in assisting the redistribution of the emitted light. The good agreement between the experimental and theoretical (linear and non-linear) results demonstrates the possibility to tailor the spectral properties of metasurfaces with an additional low-cost procedure. Based on these potential advantages, the proposed metasurface could be the object of future improvements. First of all, compared to the normal-incidence illumination method adopted to excite the optical resonances, an interesting alternative is given by in-plane illumination^63^ via the waveguiding SiO_2_ layer already built-in in the metasurface design. Within the context of integrated photonics, this strategy would effectively merge the fields of guided and free-space optics through the hybrid nature of the q-BIC mode. Moreover, in the context of all-optical infrared imaging^24^ techniques, future work may improve the spectral efficiency of the proposed metasurface by engineering additional optical resonances to further exploit FWM processes. Although a single q-BIC resonance is present for a given metasurface design^23^, the possibility to achieve multiple q-BICs in a single metasurface has been demonstrated via different approaches, such as period doubling^64^. Interestingly, the presence of multiple q-BIC resonances could also be beneficial for quantum application, such as photon pair generation via spontaneous parametric down conversion (SPDC)^65–68^. Finally, in order to achieve an on-demand tuning of the nonlinear properties, various mechanisms can be investigated, e.g., the use of active materials for all-optical refractive index modulation. Regarding the former aspect, the use of a liquid crystal cladding layer^69^ (in substitution for the proposed PMMA-based one) would result in a controllable spectral tuning of the resonances. Then, regarding all-optical refractive index modulation, a transient spectral shift of the resonances can be achieved by injecting a free-carrier density within the Si valence/conduction bands upon illumination by an ultrashort laser pulse^8^. Overall, this supports the application of HCG for the development of compact sensors and nonlinear optical devices.

## Methods

### Fabrication

All fabrication processes were carried out in a class 10-100 cleanroom environment. Initially, Si-${\langle}100{\rangle }$ silicon wafers with a thickness of 500 μm underwent standard RCA cleaning. Then the wafers were subjected to wet oxidation in a quartz tube furnace (Tempress), forming a 1.14 μm SiO_2_. Subsequently, a 489 nm thick amorphous-silicon layer was deposited through low-pressure chemical vapor deposition (LPCVD) using a Tempress furnace. The subwavelength grating structures, featuring an 840 nm pitch and a 225 nm bar width, were then defined using deep ultraviolet (DUV) lithography (Canon FPA-3000 EX4, Canon), followed by dry etching (DRIE Pegasus, SPTS Technologies Ltd.). Further details are available in Refs. ^8,32,33,70^. The PMMA layer was spun on top of the fabricating grating using a manual spin coater. The procedure involved dispensing the PMMA 495k, allowing for reflow to enter the grating structure, and then spinning at a maximum 4000 rpm speed. This resulted in a PMMA layer that is about 200 nm thick on top of the grating. The fabricated structures were examined through scanning electron microscopy (SEM Zeiss Supra 40VP, Zeiss), and shown in Fig. 2a.

### Linear spectroscopy

The refractive index of the upper a-Si layer has been obtained from ellipsometry using a VASE ellipsometer from J.A. Woollam, in the wavelength range of 500 to 1690 nm. The experimental data displayed in Fig. 2b and c were measured by using a modified version of the spectroscopy setup detailed in Ref.^8^, where we used a 200-mm (Thorlabs, LA1253) and 50-mm (Thorlabs, LA1255) focal length lens to probe the linear reflectance spectrum of the sample with and without PMMA layer, respectively.

### Non-linear spectroscopy

THG measurements have been performed with the nonlinear setup sketched in Fig. S1. The system is based on an ultrafast femtosecond laser (Coherent, Monaco) pumping an optical parametric amplifier (OPA, Coherent, Opera-F) and operating at 500 kHz repetition rate (RR). The infrared (IR) broadband (full width at half maximum ~100 nm) output beam of the OPA was spectrally tuned at around 1570 nm (see spectrum in Fig. 2b of the main text), serving as fundamental frequency (FF) excitation in the nonlinear experiment. The excitation intensity of the FF beam is controlled with half-waveplate (Thorlabs, AHWP10M-1600) and a polarizer (Thorlabs, GL10). An additional half-waveplate (Thorlabs, AHWP10M-1600) allows to finely control the FF beam polarization on the sample. To this purpose, an imaging system based on a CCD camera allows the proper monitor of the sample position. In order to achieve a loosly-collimated excitation at normal incidence, a lens (Thorlabs LA4102) focuses the FF beam on the back focal plane (BFP) of a high-numerical aperture (NA = 0.85) objective (Olympus, LCPLN100XIR) with 100x magnification power. The resulting spot size at the sample position is ${w}_{0}$ = 35 μm, with ${w}_{0}$ being the waist radius. The third-harmonic radiation emitted (back-reflected) by the sample is collected by the same objective and then directed towards the detection stage by a dichroic mirror (Thorlabs, DMLP950) placed between the first lens and the objective. A pair of lenses (Thorlabs LA1708 and LA1172) are employed to image the BFP on a CCD detector (Throlabs, CS165MU/M), thus obtaining the Fourier plane image. The properties of the emitted TH radiation are probed by a spectrometer (Andor, Kymera 193i spectrograph and iKon-M 934 CCD) and by a single photon avalanche detector (SPAD, MPD, PD-50-0TD). Residual spectral components of the FF beam are blocked before the SPAD by bandpass filter at TH wavelength. The nonlinear measurements performed in the *narrowband* regime (Fig. 3) have been accomplished by introducing bandpass filters with various central wavelength values (Thorlabs, FB15xx-12 series) in the FF beam optical path before the sample. The nonlinear measurements within the *broadband* regime (Fig. 4a) have been performed without any bandpass filter in the FF beam optical path before the sample. The spectral properties of the FF excitation reaching the sample were continuously monitored by a spectrometer (NIRQuest512). In Figs. 3 and 4 the label ${P}_{{FF}}$ denotes the incident power (average value measured by the power-meter at RR = 500 kHz) of the pulsed FF beam. The value ${P}_{{FF}}$ = 250 μW corresponds to a pulse energy ${\varepsilon }_{p}={P}_{{FF}}/{\rm{RR}}=0.5$ nJ. In Fig. 4a, the wavelength range extends above 500 nm since we introduced a long-pass filter (Thorlabs FELH0500) before the spectrometer to ensure proper spectral reference.

### Numerical Simulations

We performed numerical simulations in Comsol Multiphysics. We applied periodic Floquet boundary conditions at the unit cell, both at the fundamental and third harmonic frequencies. The input TE plane wave at the fundamental frequency is provided by periodic ports and, in the case of the simulations whose results are displayed in Figs. 2b, c, 3c, d and 4c, it impinges onto the sample at an angle ${\rm{\varphi }}$ = 0.5° from the normal. Perfectly matched layers are added to prevent unwanted reflections, the mesh size is kept smaller than 15 times the effective fundamental wavelength in the medium, and the domain size is fixed at 5.132 μm. Nonlinear currents, both at the fundamental and third harmonic frequency, are introduced. At the fundamental frequency, they are responsible for the Kerr effect and two-photon absorption, while at the third harmonic for the nonlinear frequency generation. We assumed ${{\rm{\chi }}}^{\left(3\right)}\left({\rm{\omega }}\right)=\left(7+{\rm{j}}0.8\right)\times {10}^{-20}$ m^2^/V^2^ and ${{\rm{\chi }}}^{\left(3\right)}\left(3{\rm{\omega }}\right)=\left(5+{\rm{j}}3\right)\times {10}^{-19}$ m^2^/V^2^ ^58^. We neglected the contribution to the third harmonic generation from the PMMA since ${{\rm{\chi }}}_{{\rm{Si}}}^{\left(3\right)}\gg {{\rm{\chi }}}_{{\rm{PMMA}}}^{\left(3\right)}$.

## Supplementary information

Supplementary Information for Intrapulse Multimodal Four-Wave Sum Mixing in the Visible Range from High Contrast Index Grating with PMMA layer

## Data Availability

All the data in this study are provided within the paper and its supplementary information.

## References

[1] Kamali, S. M., Arbabi, E., Arbabi, A. & Faraon, A. A review of dielectric optical metasurfaces for wavefront control. *Nanophotonics* **7**, 1041–1068 (2018).

[2] Yang, J., Gurung, S., Bej, S., Ni, P. & Lee, H. W. H. Active optical metasurfaces: comprehensive review on physics, mechanisms, and prospective applications. *Rep. Prog. Phys.* **85**, 036101 (2022). 10.1088/1361-6633/ac2aaf 35244609

[3] Kildishev, A. V., Boltasseva, A. & Shalaev, V. M. Planar photonics with metasurfaces. *Science* **339**, 1232009 (2013). 23493714 10.1126/science.1232009

[4] Yu, N. & Capasso, F. Flat optics with designer metasurfaces. *Nat. Mater.* **13**, 139–150 (2014). 24452357 10.1038/nmat3839

[5] Wang, L. et al. Nonlinear wavefront control with all-dielectric metasurfaces. *Nano Lett.* **18**, 3978–3984 (2018). 29749743 10.1021/acs.nanolett.8b01460

[6] Yu, N. et al. Light propagation with phase discontinuities: generalized laws of reflection and refraction. *Science* **334**, 333–337 (2011). 21885733 10.1126/science.1210713

[7] Kwon, H., Sounas, D., Cordaro, A., Polman, A. & Alù, A. Nonlocal metasurfaces for optical signal processing. *Phys. Rev. Lett.* **121**, 173004 (2018). 30411907 10.1103/PhysRevLett.121.173004

[8] Tognazzi, A. et al. Giant photoinduced reflectivity modulation of nonlocal resonances in silicon metasurfaces. *Adv. Photonics* **5**, 066006, 10.1117/1.AP.5.6.066006 (2023).

[9] Balthasar Mueller, J. P., Rubin, N. A., Devlin, R. C., Groever, B. & Capasso, F. Metasurface polarization optics: independent phase control of arbitrary orthogonal states of polarization. *Phys. Rev. Lett.* **118**, 113901, 10.1103/PhysRevLett.118.113901 (2017). 28368630 10.1103/PhysRevLett.118.113901

[10] Gao, Y. et al. Nonlinear holographic all-dielectric metasurfaces. *Nano Lett.* **18**, 8054–8061 (2018). 30481040 10.1021/acs.nanolett.8b04311

[11] Schulz, S. A. et al. Roadmap on photonic metasurfaces. *Appl. Phys. Lett.* **124**, 260701. 10.1063/5.0204694 (2024).

[12] Overvig, A. & Alù, A. *Laser Photonics Rev.* **16**, 2100633 (2022).

[13] Joseph, S., Pandey, S., Sarkar, S. & Joseph, J. Bound states in the continuum in resonant nanostructures: an overview of engineered materials for tailored applications. *Nanophotonics* **10**, 4175–4207 (2021).

[14] Sadrieva, Z. F. et al. Transition from optical bound states in the continuum to leaky resonances: role of substrate and roughness. *ACS Photonics* **4**, 723–727 (2017).

[15] Hsu, C. W. et al. Observation of trapped light within the radiation continuum. *Nature* **499**, 188–191 (2013). 23846657 10.1038/nature12289

[16] Wang, B. et al. Generating optical vortex beams by momentum-space polarization vortices centred at bound states in the continuum. *Nat. Photonics* **14**, 623–628 (2020).

[17] Sato, R. et al. Observation of edge bound states in the continuum at truncated silicon pillar photonic crystal. *Nat. Commun.* **15**, 10544 (2024). 39627273 10.1038/s41467-024-54929-0 PMC11615403

[18] Kodigala, A. et al. Lasing action from photonic bound states in continuum. *Nature* **541**, 196–199 (2017). 28079064 10.1038/nature20799

[19] Gentry, C. M. & Popović, M. A. Dark state lasers. *Opt. Lett.* **39**, 4136–4139 (2014). 25121670 10.1364/OL.39.004136

[20] Beliaev, L. Y., Takayama, O. & Xiao, S. Effectively detecting cardiac myoglobin by use of bound states in the continuum in silicon nitride gratings. *J. Appl. Phys.* **135**, 223101. 10.1063/5.0208969 (2024).

[21] Chen, G. Y., Li, Z. X., Chen, Y. H. & Zhang, X. D. Highly efficient polarization-entangled photon-pair generation in lithium niobate waveguides based on bound states in continuum. *Opt. Express* **29**, 12110–12123 (2021). 33984977 10.1364/OE.420792

[22] Xu, L. et al. Enhanced four-wave mixing from multi-resonant silicon dimer-hole membrane metasurfaces. *N. J. Phys.* **24**, 035002 (2022).

[23] Moretti, G. Q. et al. Si metasurface supporting multiple quasi-BICs for degenerate four-wave mixing. *Nanophotonics* **13**, 3421–3428 (2024). 39634830 10.1515/nanoph-2024-0128 PMC11501966

[24] Zheng, Z. et al. Broadband infrared imaging governed by guided-mode resonance in dielectric metasurfaces. *Light Sci. Appl.* **13**, 249 (2024). 39256381 10.1038/s41377-024-01535-w PMC11387824

[25] Chang-Hasnain, C. J. & Yang, W. High-contrast gratings for integrated optoelectronics. *Adv. Opt. Photon.* **4**, 379 (2012).

[26] Finco, G. et al. Guided-mode resonance on pedestal and half-buried high-contrast gratings for biosensing applications. *Nanophotonics* **10**, 4289–4296 (2021).

[27] Armstrong, J. A., Bloembergen, N., Ducuing, J. & Pershan, P. S. Interactions between Light Waves in a Nonlinear Dielectric. *Phys. Rev.* **127**, 1918–1939 (1962).

[28] Koshelev, K. et al. Nonlinear metasurfaces governed by bound states in the continuum. *ACS Photonics* **6**, 1639–1644 (2019).

[29] Zograf, G. et al. High-harmonic generation from resonant dielectric metasurfaces empowered by bound states in the Continuum. *ACS Photonics* **9**, 567–574 (2022).

[30] McDonnell, C., Deng, J., Sideris, S., Ellenbogen, T. & Li, G. Functional THz emitters based on Pancharatnam-Berry phase nonlinear metasurfaces. *Nat. Commun.* **12**, 30 (2021). 33397951 10.1038/s41467-020-20283-0 PMC7782718

[31] Schlickriede, C. et al. Nonlinear imaging with all-dielectric metasurfaces. *Nano Lett.* **20**, 4370–4376 (2020). 32374616 10.1021/acs.nanolett.0c01105

[32] Beliaev, L. Y. et al. Pedestal high-contrast gratings for biosensing. *Nanomaterials* **12**, 1748 (2022). 35630973 10.3390/nano12101748 PMC9145707

[33] Franceschini, P. et al. Nonlocal resonances in pedestal high-index-contrast metasurfaces based on a silicon-on-insulator platform. *Appl. Phys. Lett.* **123**, 071701. 10.1063/5.0159275 (2023).

[34] Hail, C. U., Michaeli, L. & Atwater, H. A. Third harmonic generation enhancement and wavefront control using a local High-QMetasurface. *Nano Lett.* **24**, 2257–2263 (2024). 38346272 10.1021/acs.nanolett.3c04476

[35] Colom, R. et al. Enhanced four-wave mixing in doubly resonant Si nanoresonators. *ACS Photonics* **6**, 1295–1301 (2019).

[36] Liu, T., Qin, M., Wu, F. & Xiao, S. High-efficiency optical frequency mixing in an all-dielectric metasurface enabled by multiple bound states in the continuum. *Phys. Rev. B* **107**, 075441. 10.1103/PhysRevB.107.075441 (2023).

[37] Grinblat, G., Li, Y., Nielsen, M. P., Oulton, R. F. & Maier, S. A. Degenerate Four-wave mixing in a multiresonant germanium nanodisk. *ACS Photonics* **4**, 2144–2149 (2017).

[38] Jin, B. & Argyropoulos, C. Enhanced four-wave mixing with nonlinear plasmonic metasurfaces. *Sci. Rep.* **6**, 28746. 10.1038/srep28746 (2016). 27345755 10.1038/srep28746 PMC4921841

[39] Moretti, G. Q., Cortés, E., Maier, S. A., Bragas, A. V. & Grinblat, G. Engineering gallium phosphide nanostructures for efficient nonlinear photonics and enhanced spectroscopies. *Nanophotonics* **10**, 4261–4271 (2021).

[40] Colman, P. et al. Observation of parametric gain due to four-wave mixing in dispersion engineered GaInP photonic crystal waveguides. *Opt. Lett.* **36**, 2629–2631 (2011). 21765490 10.1364/OL.36.002629

[41] Liu, S. et al. An all-dielectric metasurface as a broadband optical frequency mixer. *Nat. Commun.* **9**, 2507 (2018). 29955051 10.1038/s41467-018-04944-9 PMC6023909

[42] Li, G. et al. *Laser Photonics Rev.* **12**, 1800034. 10.1002/lpor.201800034 (2018).

[43] Dai, Y. et al. Broadband Plasmon-enhanced four-wave mixing in monolayer MoS_2_. *Nano Lett.* **21**, 6321–6327 (2021). 34279968 10.1021/acs.nanolett.1c02381 PMC8323120

[44] Wang, S. S. & Magnusson, R. Theory and applications of guided-mode resonance filters. *Appl. Opt.* **32**, 2606–2613 (1993). 20820422 10.1364/AO.32.002606

[45] Gambino, F., Giaquinto, M., Ricciardi, A. & Cusano, A. (INVITED) A review on dielectric resonant gratings: Mitigation of finite size and Gaussian beam size effects. *Results Opt* **6**, 100210, 10.1016/j.rio.2021.100210 (2022).

[46] Ali, U., Karim, K. J. B. A. & Buang, N. A. a review of the properties and applications of poly (Methyl Methacrylate) (PMMA). *Polym. Rev* **55**, 678–705 (2015).

[47] D’Amore, F., Lanata, M., Pietralunga, S. M., Gallazzi, M. C. & Zerbi, G. Enhancement of PMMA nonlinear optical properties by means of a quinoid molecule. *Opt. Mater.* **24**, 661–665 (2004).

[48] W. Demtröder, Laser Spectroscopy - Basic Concepts and Instrumentation (Springer, 2002).

[49] Bulgakov, E. N. & Sadreev, A. F. Bloch bound states in the radiation continuum in a periodic array of dielectric rods. *Phys. Rev. A* **90**, 053801. 10.1103/PhysRevA.90.053801 (2014).

[50] Gao, X. et al. Formation mechanism of guided resonances and bound states in the continuum in photonic crystal slabs. *Sci. Rep.* **6**, 31908. 10.1038/srep31908 (2016). 27557882 10.1038/srep31908 PMC4997268

[51] Koshelev, K., Lepeshov, S., Liu, M., Bogdanov, A. & Kivshar, Y. Asymmetric metasurfaces with high-Q resonances governed by bound states in the continuum. *Phys. Rev. Lett.* **121**, 193903 (2018). 30468599 10.1103/PhysRevLett.121.193903

[52] Jin, J. et al. Topologically enabled ultrahigh-Q guided resonances robust to out-of-plane scattering. *Nature* **574**, 501–504 (2019). 31645728 10.1038/s41586-019-1664-7

[53] Tognazzi, A. et al. Third-harmonic light polarization control in magnetically resonant silicon metasurfaces. *Opt. Express* **29**, 11605–11612, 10.1364/OE.419829 (2021). 33984937 10.1364/OE.419829

[54] Gili, V. F. et al. Monolithic AlGaAs second-harmonic nanoantennas. *Opt. Express* **24**, 15965–15971 (2016). 27410864 10.1364/OE.24.015965

[55] Sinev, I. S. et al. Observation of ultrafast self-action effects in quasi-BIC resonant metasurfaces. *Nano Lett.* **21**, 8848–8855 (2021). 34633185 10.1021/acs.nanolett.1c03257

[56] R. W. Boyd, Nonlinear Optics, Nonlinear Optics (Academic Press, 2003), 371-407.

[57] Fano, U. Effects of configuration interaction on intensities and phase shifts. *Phys. Rev.* **124**, 1866–1878 (1961).

[58] Scalora, M. et al. Resonant, broadband, and highly efficient optical frequency conversion in semiconductor nanowire gratings at visible and UV wavelengths. *J. Opt. Soc. Am. B* **36**, 2346 (2019).

[59] Kurvits, J. A., Jiang, M. & Zia, R. Comparative analysis of imaging configurations and objectives for Fourier microscopy. *J. Opt. Soc. Am. A* **32**, 2082–2092 (2015). 10.1364/JOSAA.32.002082 26560923

[60] Minovich, A. E. et al. Functional and nonlinear optical metasurfaces. *Laser Photonics Rev.* **9**, 195 (2015).

[61] Li, G., Zhang, S. & Zentgraf, T. Nonlinear photonic metasurfaces. *Nat. Rev. Mater.* **2**, 17010. 10.1038/natrevmats.2017.10 (2017).

[62] Chen, S., Li, G., Cheah, K. W., Zentgraf, T. & Zhang, S. Controlling the phase of optical nonlinearity with plasmonic metasurfaces. *Nanophotonics* **7**, 1013–1024 (2018).

[63] Huang, H. et al. Leaky-wave metasurfaces for integrated photonics. *Nat. Nanotechnol.* **18**, 580–588 (2023). 37157023 10.1038/s41565-023-01360-z

[64] Overvig, A., Shrestha, S. & Yu, N. Dimerized high contrast gratings. *Nanophotonics* **7**, 1157–1168 (2018).

[65] Parry, M. et al. Enhanced generation of nondegenerate photon pairs in nonlinear metasurfaces. *Adv. Photonics* **3**, 055001 (2021).

[66] Santiago-Cruz, T. et al. Photon pairs from resonant metasurfaces. *Nano Lett.* **21**, 4423–4429 (2021). 33971095 10.1021/acs.nanolett.1c01125 PMC8289292

[67] Sharapova, P. R., Kruk, S. S. & Solntsev, A. S. *Laser Photonics Rev.* **17**, 2200408 (2023).

[68] Jiang, Y., Zhang, J., Ma, J., Neshev, D. N. & Sukhorukov, A. A. Flatband nonlinear metasurface for broad-angle photon-pair generation. *APL Quantum* **2**, 026115. 10.1063/5.0247669 (2025).

[69] Cort, W. D., Beeckman, J., Claes, T., Neyts, K. & Baets, R. Wide tuning of silicon-on-insulator ring resonators with a liquid crystal cladding. *Opt. Lett.* **36**, 3876–3878 (2011). 21964127 10.1364/OL.36.003876

[70] Beliaev, Y. et al. Optical biosensors based on nanostructured silicon high-contrast gratings for Myoglobin detection. *ACS Appl. Nano Mater.* **6**, 12364–12371 (2023).

