# Supplementary material for: Intrapulse multimodal four-wave sum mixing in the visible range from high contrast index grating with PMMA layer
Source: Light Sci Appl. 2026 Jan 5;15:51. doi: 10.1038/s41377-025-02090-8 (PMC12765861; doi:10.1038/s41377-025-02090-8)
Supplement: Supplementary file 1 — Supplementary Information for Intrapulse Multimodal Four-Wave Sum Mixing in the Visible Range from High Contrast Index Grating with PMMA layer [file 41377_2025_2090_MOESM1_ESM.pdf]

Supplementary Information for Intrapulse Multimodal Four-Wave Sum Mixing in the Visible Range from High Contrast Index Grating with PMMA layer

Paolo Franceschini,[1,2] Andrea Tognazzi,[2,3] Evgenii Menshikov,[1,4] Leonid Y. Beliaev,[5] Radu Malureanu,[5,6] Osamu Takayama,[5] Ivano Alessandri,[1,2] Alfonso Carmelo Cino,[3] Domenico de Ceglia,[1,2] Andrei V. Lavrinenko,[5] and Costantino De Angelis[1,2,*]

[1] *University of Brescia, Department of Information Engineering, Via Branze 38, 25123, Brescia, Italy*

[2] *National Institute of Optics – National Research Council (INO-CNR), Via Branze 45, 25123, Brescia, Italy*

[3] *University of Palermo, Department of Engineering, Viale delle Scienze, 90128,Palermo, Italy*

[4] *School of Physics and Engineering, ITMO University, Kronverksky Pr. 49, bldg. A, St. Petersburg, 197101, Russia*

[5] *Department of Electrical and Photonics Engineering, Technical University of Denmark, Ørsteds Plads, Building 345A, DK-2800 Kongens Lyngby, Denmark.*

[6] *DTU Nanolab, National Centre for Nano Fabrication and Characterization, Building 347, DK-2800 Kongens Lyngby, Denmark.*

CONTENTS

I. Additional Notes on the Experimental Setup S3
A. Sketch of the Nonlinear and Fourier Microscopy Setup S3
B. Notes on the Measurement of THG count rate. S4

II. Additional Experimental Data S5
A. Linear Spectroscopy Measurements S5
B. Nonlinear Spectroscopy Measurements S6
C. Nonlinear Fourier Microscopy S7
D. Analysis of Nonlinear Spectra - Narrowband Regime S8
E. THG Efficiency S9

III. Additional Numerical Results S10
A. Mode Spatial Distribution S10
B. Mode Dispersion S13
C. Effect of Sidewall Tapering S15

IV. Additional Notes on Numerical Simulations S17
A. Nonlinear Problem - Narrowband Regime S17
B. Nonlinear Problem - Kerr effect and Two-photon absorption S18
C. Nonlinear Problem - Effective model for FWM S19
D. TH Emission through Diffraction Orders S20

References S21

* costantino.deangelis@unibs.it

I. ADDITIONAL NOTES ON THE EXPERIMENTAL SETUP

A. Sketch of the Nonlinear and Fourier Microscopy Setup

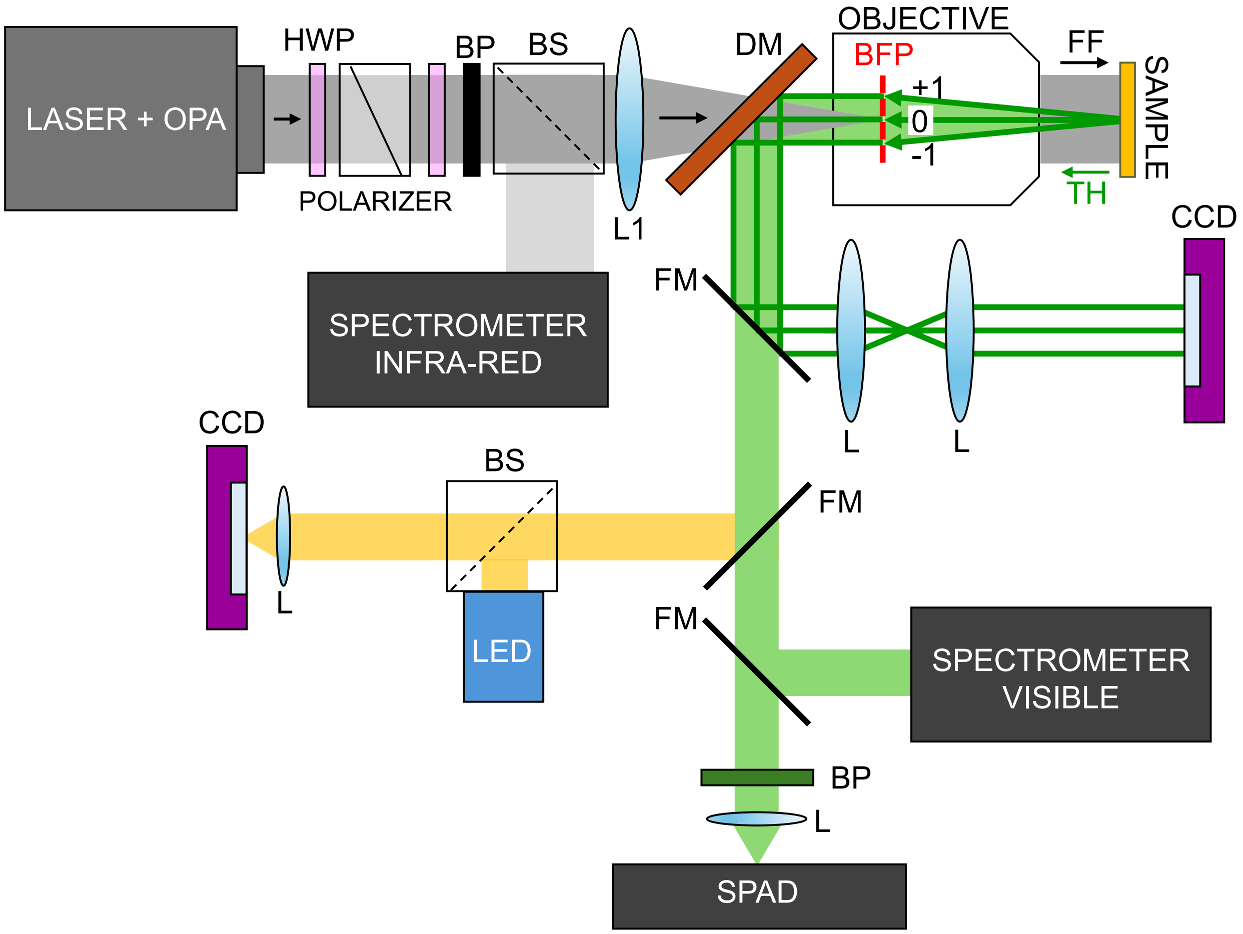

FIGURE S1. **Experimental Setup (Non-linear).** OPA: optical parametric amplifier; HWP: half-wave plate; BP: band-pass spectral filter; L: lens; DM: dichroic mirror; BFP: back focal plane; FF: fundamental frequency beam; TH: third-harmonic beam; FM: flip mirror; BS: beam-splitter; SPAD: single photon avalanche detector.

B. Notes on the Measurement of THG count rate.

In Fig. 3a of the main text the reported TH intensity count rate has been measured by the SPAD, which has a dark count rate (due to thermal noise) around 40 cts s^{-1} (with a standard deviation value of 5-10 cts s^{-1}). When processing the data, the background due to thermal noise is subtracted from the raw data thus to obtain the net intensity count rate (as those shown in Fig. 3a of the main text). In the case of the experimental data measured at λ_{FF}=1600 nm, the raw THG signal measured at low-level power ($P_{\mathrm{FF}} \sim 0.01$ mW) corresponds to the thermal noise. Therefore, the deviation of the experimental data from the fit represents the standard deviation value of the dark count rate.

II. ADDITIONAL EXPERIMENTAL DATA

A. Linear Spectroscopy Measurements

Figure S2 shows the measured linear reflectance spectra of the PMMA sample (blue solid line) in the case of a transverse magnetic (TM) probe light (*i.e.*, electric field perpendicular to the bars). The sharp spectral feature is ascribed to mode BM_1 (see main text for details). The red solid line displays the optimized simulated reflectance spectrum calculated by assuming the same values of the geometrical parameters as in Fig. 2a of the main text.

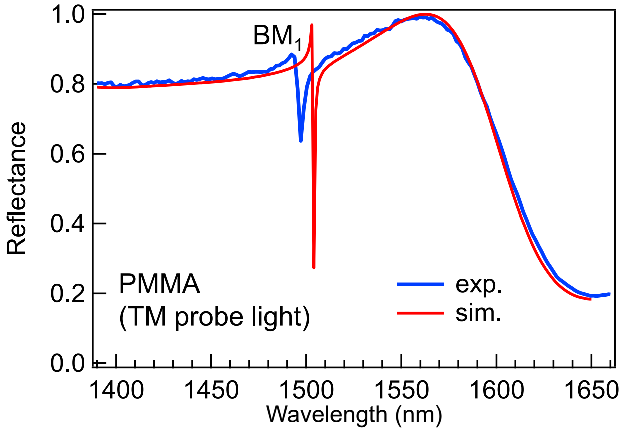

FIGURE S2. **Linear Characterization TM Mode.** Measured (blue solid line) and calculated (red solid line) reflectance spectra of the metasurface (sample with PMMA layer) measured with TM probe light.

B. Nonlinear Spectroscopy Measurements

Figure S3 shows the measured TH generation intensity from the PMMA sample as a function of the FF beam polarization angle θ in the case of a narrow band ($\Delta\lambda_{\mathrm{FF}} = 12$ nm) excitation at $\lambda_{\mathrm{FF}} = 1590$ nm.

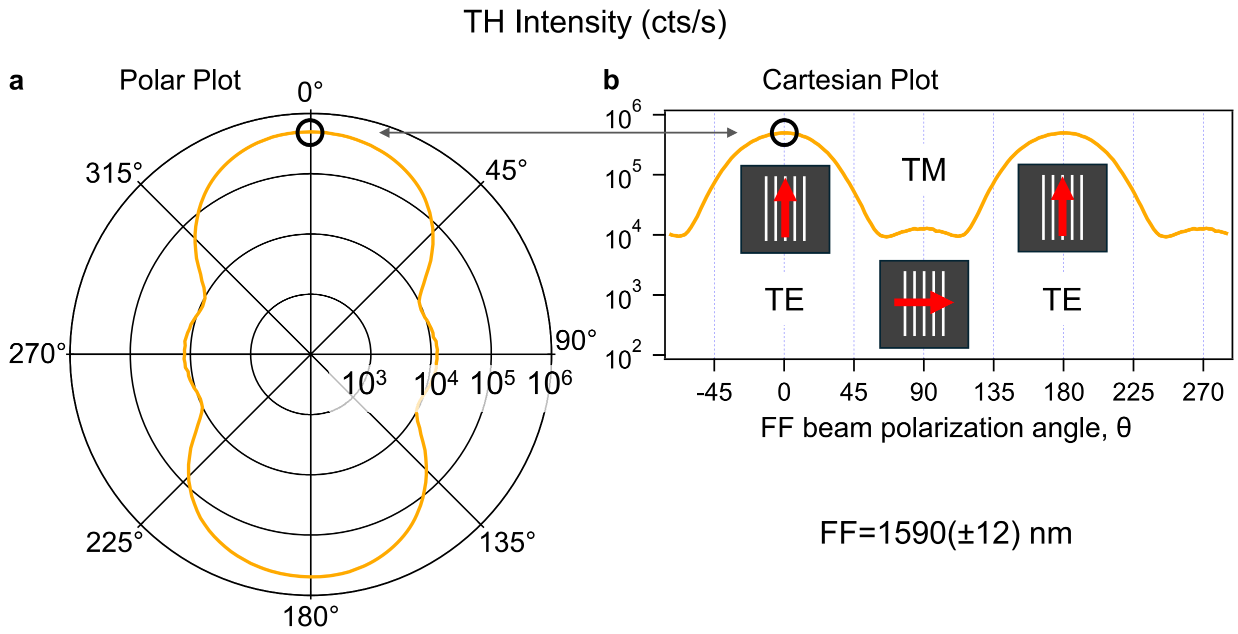

FIGURE S3. **Polarization-resolved THG.** Measured THG intensity as a function of the FF beam polarization angle: (a) polar plot and (b) cartesian plot. TE mode at 0° and TM mode at 90°. The mesurement has been performed with $P_{\mathrm{FF}} = 250$ μW at 500 kHz repetition rate.

C. Nonlinear Fourier Microscopy

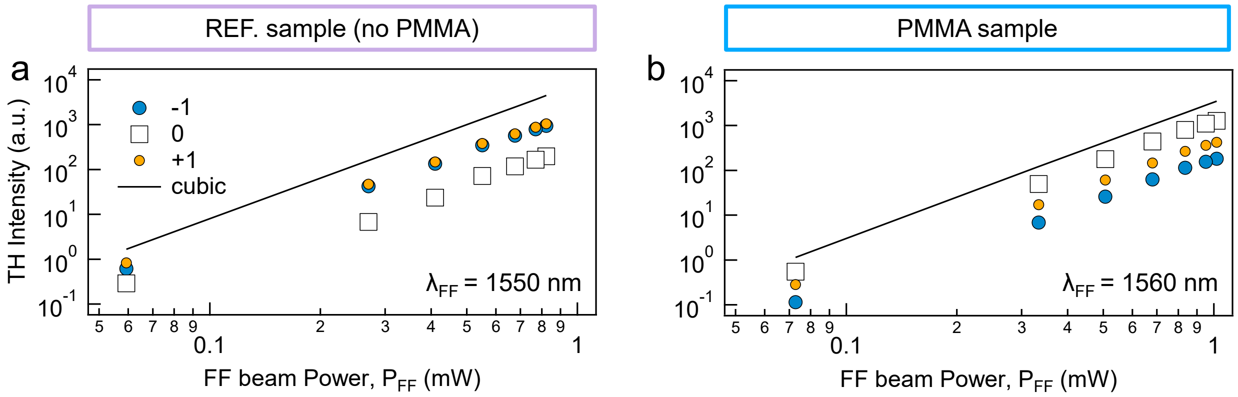

FIGURE S4. **Power-dependent TH Emission through Diffraction Orders.** Experimental intensity of the TH radiation emitted trough diffraction channels of order 0th (white square markers) and ±1 (yellow and light blue circulr markers, respectively) as a function of the FF excitation power for the sample without (panel a, $\lambda_{\mathrm{FF}} = 1550$ nm) and with (panel b, $\lambda_{\mathrm{FF}} = 1560$ nm) PMMA layer.

D. Analysis of Nonlinear Spectra - Narrowband Regime

Starting from a FF electric field whose spectrum (in frequency domain $\nu = c/\lambda$) is described as a Gassian profile of the form

$$E_{\mathrm{FF}}(\nu) \propto \exp\left[-\left(\frac{\nu-\nu_{\mathrm{FF}}}{w}\right)^2\right], \tag{S1}$$

with ν_{FF} and w being the central frequency and bandwidth, the resulting TH polarization (which is proportional to the TH electric field) can be calculated [1] as the convolution

$$P_{\mathrm{TH}}(\nu) \propto E_{\mathrm{FF}}(\nu) * E_{\mathrm{FF}}(\nu) * E_{\mathrm{FF}}(\nu)\,. \tag{S2}$$

In particular, in Eq. (S1), the bandwidth is defined such that $w = \Delta\nu_{\mathrm{FF}}/\sqrt{2\log 2}$, with $\Delta\nu_{\mathrm{FF}}$ being the full width at half maximum (FWHM) of the spectral intensity profile $I_{\mathrm{FF}}(\nu)$ of the FF excitation (in frequency domain). Therefore, from Eq. (S2), the spectral region at TH frequency is described as

$$E_{\mathrm{TH}}(\nu) \propto \exp\left[-\left(\frac{\nu-3\nu_{\mathrm{FF}}}{\sqrt{3}\,w}\right)^2\right]. \tag{S3}$$

Since the intensity is proportional to the square of the electric field, it follows that

$$I_{\mathrm{FF}}(\nu) \propto \left|E_{\mathrm{FF}}(\nu)\right|^2 \propto \exp\left[-\left(\frac{\nu-\nu_{\mathrm{FF}}}{w/\sqrt{2}}\right)^2\right] \tag{S4}$$

and

$$I_{\mathrm{TH}}(\nu) \propto \exp\left[-\left(\frac{\nu-3\nu_{\mathrm{FF}}}{w\cdot\sqrt{3/2}}\right)^2\right]. \tag{S5}$$

In the case of a narrowband spectral profile, the following relation holds

$$\Delta\nu_{\mathrm{FF}} = \frac{c\cdot\Delta\lambda_{\mathrm{FF}}}{\lambda_{\mathrm{FF}}^2},$$

with $\lambda_{\mathrm{FF}} = c/\nu_{\mathrm{FF}}$ and $\Delta\lambda_{\mathrm{FF}}$ being the FWHM of the spectral intensity profile $I_{\mathrm{FF}}(\lambda)$ of the FF excitation (in wavelength domain). Therefore, considering the experimental condition adopted for the measurement displayed in Fig. 3b (main text), the expected bandwidth of the TH spectrum obtained for a FF excitation with $\lambda_{\mathrm{FF}} = 1590$ nm and $\Delta\lambda_{\mathrm{FF}} = (11.6 \pm 0.1)$ nm is $\Delta\lambda_{\mathrm{TH}} = (2.23 \pm 0.02)$ nm. By fitting a Gaussian profile to the experimental data in Fig. 3b (main text), we obtain $\Delta\lambda_{\mathrm{TH}} = (2.14 \pm 0.03)$ nm, which is consistent with the expected value.

E. THG Efficiency

THG efficiency has been calculated from experimental data as

$$\eta_{\mathrm{TH}} = \frac{I_{\mathrm{TH}} \cdot E_{\mathrm{ph,TH}}}{P_{\mathrm{FF}}}, \tag{S6}$$

where I_{TH} and $E_{\mathrm{ph,TH}}$ are the intensity (expressed as counts per second) and the photon energy of the TH radiation, respectively. From Eq. (S6), the TH efficiency values reported in Table S.I have been calculated as

$$\eta_{\mathrm{TH}} = \frac{I_{\mathrm{TH}}[\mathrm{cts\ s^{-1}}] \cdot (1239.84/\lambda_{\mathrm{TH}}[\mathrm{nm}]) \cdot 1.602 \cdot 10^{-19}}{P_{\mathrm{FF}}[\mu\mathrm{W}] \cdot 10^{-6}}. \tag{S7}$$

TABLE S.I. Intensity of the TH radiation emitted at resonant excitation of the BE_1 mode with $P_{\mathrm{FF}} = 250\ \mu$W (see Figures 3c and d of the main text).

Sample	λ_{FF} [nm]	λ_{TH} [nm]	I_{TH} [cts s^{-1}]	η_{TH}
REF (no PMMA layer)	1570	523	$6.25 \cdot 10^6$	$9.5 \cdot 10^{-9}$
PMMA (with PMMA layer)	1590	530	$6.00 \cdot 10^5$	$9.0 \cdot 10^{-10}$

III. ADDITIONAL NUMERICAL RESULTS

A. Mode Spatial Distribution

In this section, we detail the polarization vector of the electric and magnetic field components of the resonant modes supported by the REF and PMMA samples calculated by eigenmode analysis (Fig. S5 and S6, respectively). For clarity, the field distribution of the modes is also shown (colorscale).

We clarify that:

- For the TE modes, *i.e.*, BE_1 and LE_1, the electric field is polarized along the out-of-plane direction (y-axis), while the magnetic field component lies in-plane (xz-plane).
- For the TM modes, the magnetic field is polarized in the out-of-plane direction, while the electric field lies in-plane.

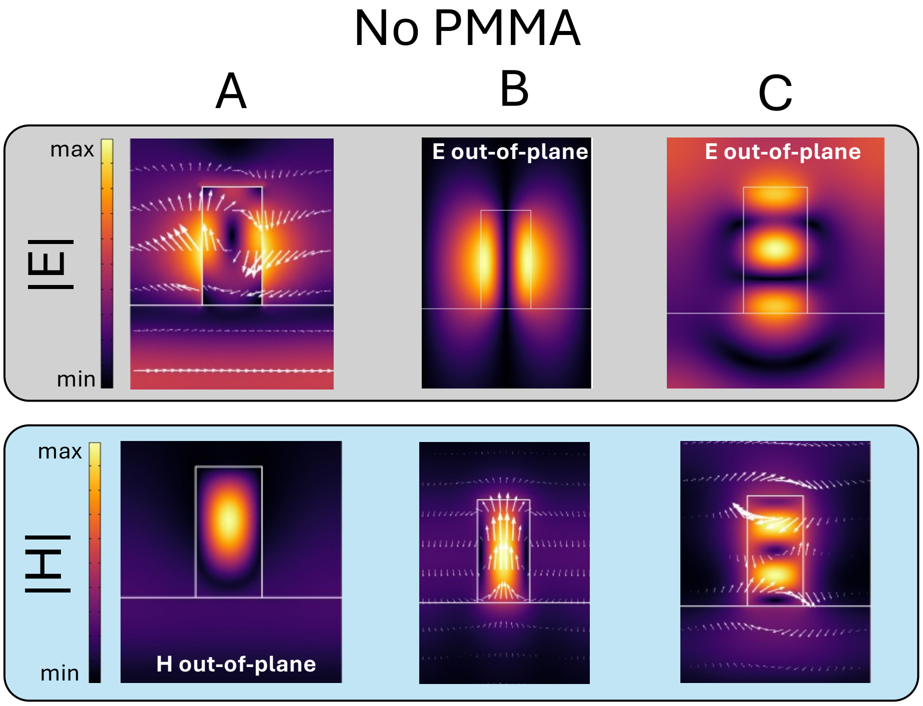

FIGURE S5. **Mode Spatial Distribution (REF sample).** Electric (top row) and magnetic (bottom row) field distribution obtained from the eigenmode analysis for the REF sampe (no PMMA layer). Arrows represent the orientation of the in-plane electric/magnetic field components. Arrows are not represented when the field is completely polarized in the out-of-plane direction.

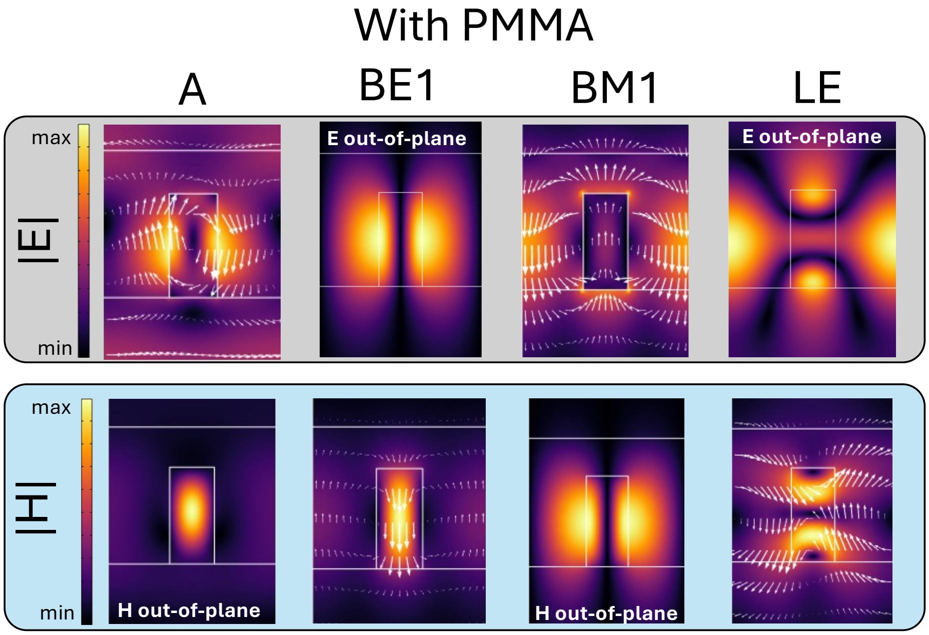

FIGURE S6. **Mode Spatial Distribution (PMMA sample).** Electric (top row) and magnetic (bottom row) field distribution obtained from the eigenmode analysis for the sampe with PMMA layer. Arrows represent the orientation of the in-plane electric/magnetic field components. Arrows are not represented when the field is completely polarized in the out-of-plane direction.

B. Mode Dispersion

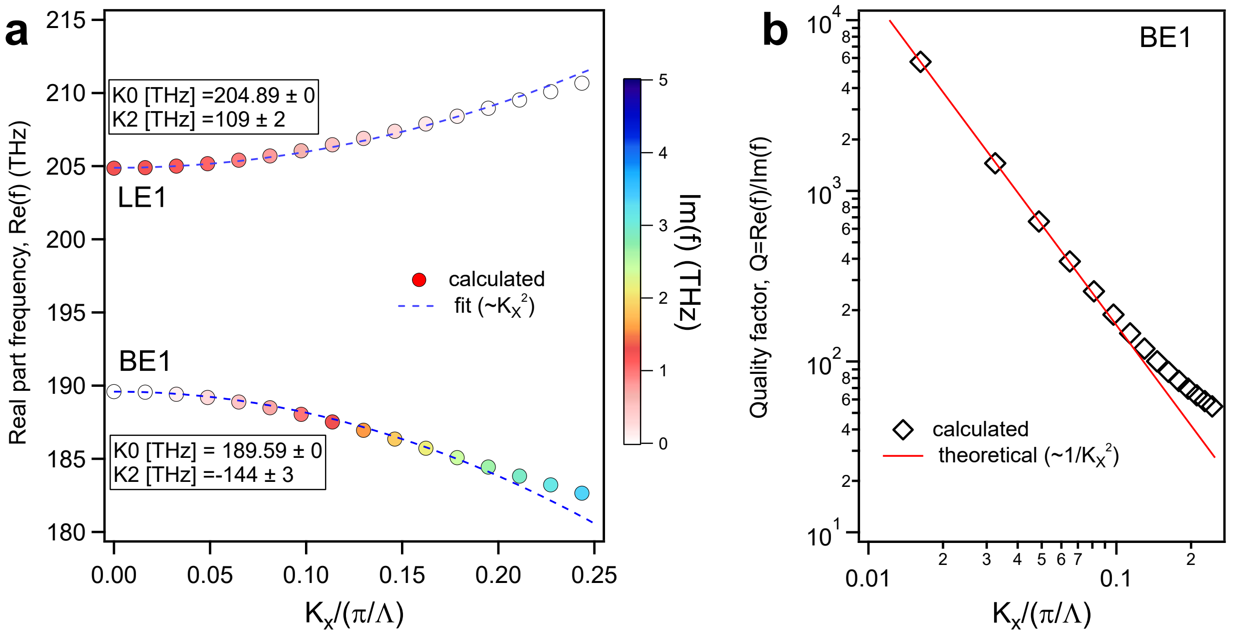

FIGURE S7. **LE and BE1 Mode Dispersion (PMMA sample).** (a) Band diagram (real part of eigenfrequency, Re(f), as a function of the in-plane component of the normalized Bloch wave vector, $K_x/(\pi/\Lambda)$) for the TE modes of the sample with PMMA layer obtained from the eigenvalue solver in Comsol (circles). The blue dashed lines are obtained by fitting a parabolic function $f(x) = K0 + K2 \cdot x^2$ to the calculated mode dispersion. For simplicity, as in the main text, we neglected the material dispersion. The lower branch is the BE1 mode while the upper branch is the LE1 mode. (b) Quality factor Q of the BE$_1$ mode (black markers) as a function of the in-plane component of the normalized Bloch wave vector $K_x/(\pi/\Lambda)$ retrieved from the data in panel a. The red line denotes a theoretical quadratically decaying profile.

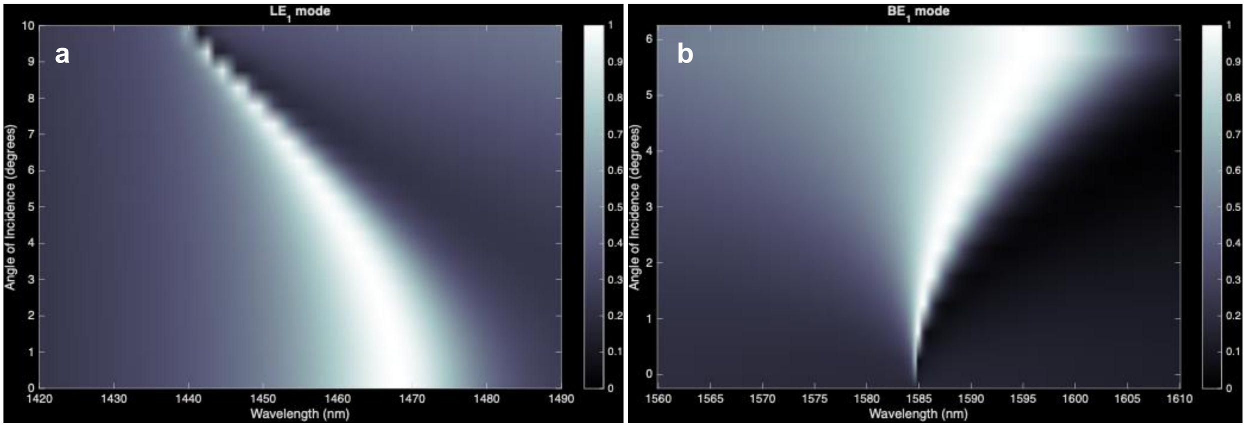

FIGURE S8. **Reflectivity close to resonant modes (PMMA sample).** Reflectivity as a function of the wavelength (horizontal axis) and impinging angle (vertical axis) for TE polarization close to LE_1 (a) and BE_1 (b) modes. The linear spectra includes the material dispersion.

C. Effect of Sidewall Tapering

In this section, we numerically investigate the effect of sidewall tapering on the Q-factor of the BE_1 mode resonance profile. In particular, we simulated the linear properties of the metasurface by assuming a trapezoidal cross section of the grating bar. As shown in the inset Fig. S9, the shape of the cross section is parametrized by an angle θ, where a positive (negative) value of θ denotes the top being shorter (larger) than the bottom (the case θ=0° corresponds to a rectangular cross section of the bar). The value of the top side was kept fixed at w=248 nm. The calculated linear reflectance spectra shown in Fig. S9 clearly reveal that the spectral feature ascribed to BE_1 mode red-shifts when moves from negative to positive values. In order to investigate the modulation of the Q-factor resulting from sidewall tapering, we retrieve the value of the Q-factor for different values of by fitting a theoretical Fano lineshape [2]

$$f(\hbar\omega) \propto \frac{(q \cdot \Gamma/2 + \hbar\omega - E_0)^2}{(\Gamma/2)^2 + (\hbar\omega - E_0)^2} \tag{S8}$$

- where $\omega = 2\pi c/\lambda$, q is the profile index, Γ is the linewidth of the resonance (corresponding to the full-width at half maximum of the limit as Lorenzian-profile), and E_0 is the spectral position of the resonance - to the calculated curves in Fig. S9. The results obtained from the analysis are shown in Tab. S.II.

TABLE S.II. Values of the Q-factor of the BE_1 mode resonance for different values of the sidewall angle θ retrieved from the analysis of the profiles in Fig. S9.

Sidewall angle θ	$Q = E_0/\Gamma$
-0.50°	$\sim 1.4 \cdot 10^4$
-0.25°	$\sim 1.4 \cdot 10^4$
0.00°	$\sim 6.2 \cdot 10^4$
+0.25°	$\sim 1.4 \cdot 10^4$
+0.50°	$\sim 1.4 \cdot 10^4$

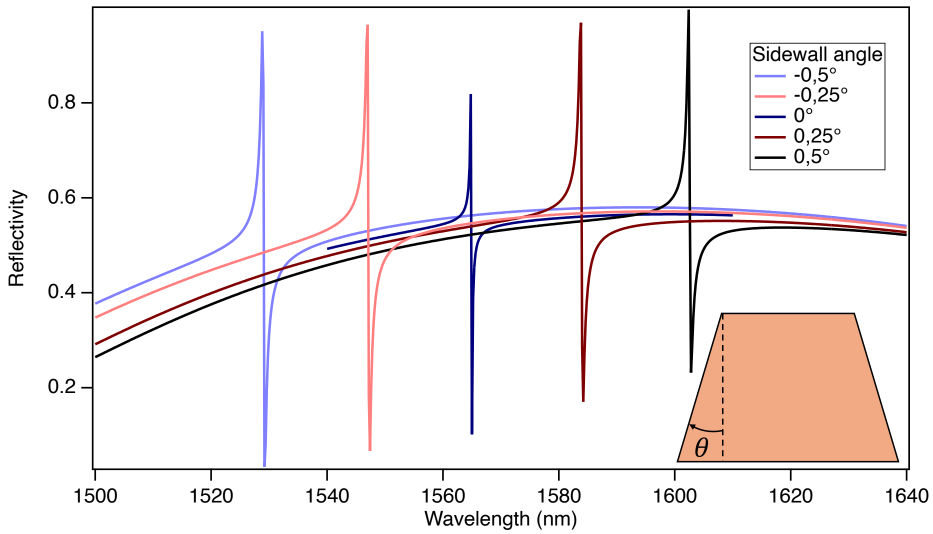

FIGURE S9. **Effect of Sidewall Tapering.** Reflectivity spectra as a function of the wavelength fordifferent sidewall angles for the REF sample. For the positive sidewall angles the bottom base is larger than the top one. A sidewall angle of 0.5° corresponds to an increase of $\sim$ 8.5 nm of the bottom base width. Negative angles corresponds to a smaller bottom base.

IV. ADDITIONAL NOTES ON NUMERICAL SIMULATIONS

A. Nonlinear Problem - Narrowband Regime

Here, we describe the procedure adopted to calculate the TH intensity profiles displayed in Figures 3c and d of the main text. In brief, to mimic the effect of the bandpass filters with finite width employed in the experiments, we solved the numerical model in a 20 nm range around the central frequency of the filter. We assumed a gaussian distribution of the field intensity, with standard deviation 6 nm, and integrated the third harmonic power over the whole frequency range. The step by step procedure can be described as follow:

- We set the values of λ_{FF}, $\Delta\lambda_{\text{FF}}$, and I_{FF}, which represent the central wavelength, the bandwidth (full width at half maximum), and intensity of the FF excitation beam.
- We set the interval in which the single-frequency components (indexed by q) of the FF excitation lay: $\Gamma_{\text{FF}} = \{\lambda_{\text{FF}}[\text{nm}] - 10; \lambda_{\text{FF}}[\text{nm}] + 10\}$.
- We set the spectral distance between two consecutive single-frequency components: $\delta\lambda = \lambda_{q+1} - \lambda_q = 0.1$ nm, where λ_q is the spectral position of the q-th single-frequency component.
- Given Γ_{FF} and $\delta\lambda$, the total number of components included in the FF beam is

$$N_{\text{com}} = \frac{(\lambda_{\text{FF}}[\text{nm}] + 10) - (\lambda_{\text{FF}}[\text{nm}] - 10)}{\delta\lambda} + 1 = 201$$

  and each component is located at

$$\lambda_q[\text{nm}] = \lambda_{\text{FF}}[\text{nm}] - 10 + q \cdot \delta\lambda, \qquad \text{with} \qquad q = 0, \ldots, N_{\text{com}}.$$

- The deposited power on the excitation port is calculated starting from the intensity of the q-th component. The intensity (I_q) is calculated by assuming a Gaussian profile of the spectrum of the FF excitation:

$$I_q = \mathcal{I}_0 \cdot \exp\left[-\frac{4 \cdot \log 2}{\Delta\lambda_{\text{FF}}^2} \cdot (\lambda_{\text{FF}} - \lambda_q)^2\right],$$

  where $\mathcal{I}_0$ is the maximum intensity.

- Once the inputs for the q-th component is fully characterized, we numerically solve the problem for a plane wave exciting the structure at fundamental frequency $\nu_q = c/\lambda_q$. By computing the flux of the Poyinting vector we obtain the third harmonic power ($P_{\mathrm{TH}}^{(q)}$) of the radiation emitted at the TH frequency $3\nu_q$.

- The power of the total radiation emitted upon narrowband excitation is calculated as $P_{\mathrm{TH}} = \sum_{q=0}^{N} P_{\mathrm{TH}}^{(q)}$.

B. Nonlinear Problem - Kerr effect and Two-photon absorption

The results obtained in Figure 4c of the main paper are obtained by performing a parametric sweep over the frequency range and assuming a continuous wave excitation. The distortion in the spectra for increasing pump power, which ultimately lead to instability, is due to the nonlinear currents at the fundamental frequency, which are related to the $\chi^{(3)}(\omega)$ term.

C. Nonlinear Problem - Effective model for FWM

Figure 4b of the main text shows the third-order polarization at the TH frequency $\left|P^{(3)}(\omega)\right|^2 = \left|\mathcal{F}\left\{P^{(3)}_{\text{eff}}(t)\right\}(\omega)\right|^2$, where the effective third-order polarization in the time domain has been calculated as $P^{(3)}_{\text{eff}}(t) \propto \left[\mathcal{F}^{-1}\left\{t_{\text{eff}}(\omega)\cdot E_{\text{FF}}(\omega)\right\}(t)\right]^3$. In particular,

- A Gaussian profile was assumed for the the broadband spectrum of the electric field at FF ($E_{FF}(\omega)$). The spectral width of the FF beam has been retrieved by fitting a Gaussian profile to the intensity spectrum of the FF beam in Fig. 2b of the main text.
- The effective linear response function of the metasurface $t_{\text{eff}}(\omega)$ has been modeled, starting from the reflectance spectrum in Fig. 2b of the main text, as

$$t_{\text{eff}}(\omega) = f_{\text{BE1}}(\omega) + f_{\text{LE1}}(\omega) + b(\omega)\,, \tag{S9}$$

  where $f_{\text{X}}(\omega)$ (with X=BE1, LE1) is a Fano profile (as the one in Eq. S8) and $b(\omega)$ represents the slowly-varying background.

D. TH Emission through Diffraction Orders

The intensity of each diffraction order is obtained by performing the Fast Fourier Transform (FFT) of the electric field in the unit cell at a fixed height above the silicon grating. The height value is 253 nm above the silicon bars for both the gratings with and without the PMMA layers. We performed a parametric sweep over the whole frequency range assuming continuous wave excitation with the same pump intensity and computed the FFT for wavelengths in the range 1530-1610 nm.

Here, we report the intensity of the TH radiation emitted through the diffraction channel of order 0 and +1. We note that orders ±1 completely overlap in the simulations. In particular, Fig. S10 shows the calculated intensity profiles $I_{\mathrm{TH}}^{(m)}$ of the m-th order ($m = 0, 1$) resulting from single-frequency numerical simulations.

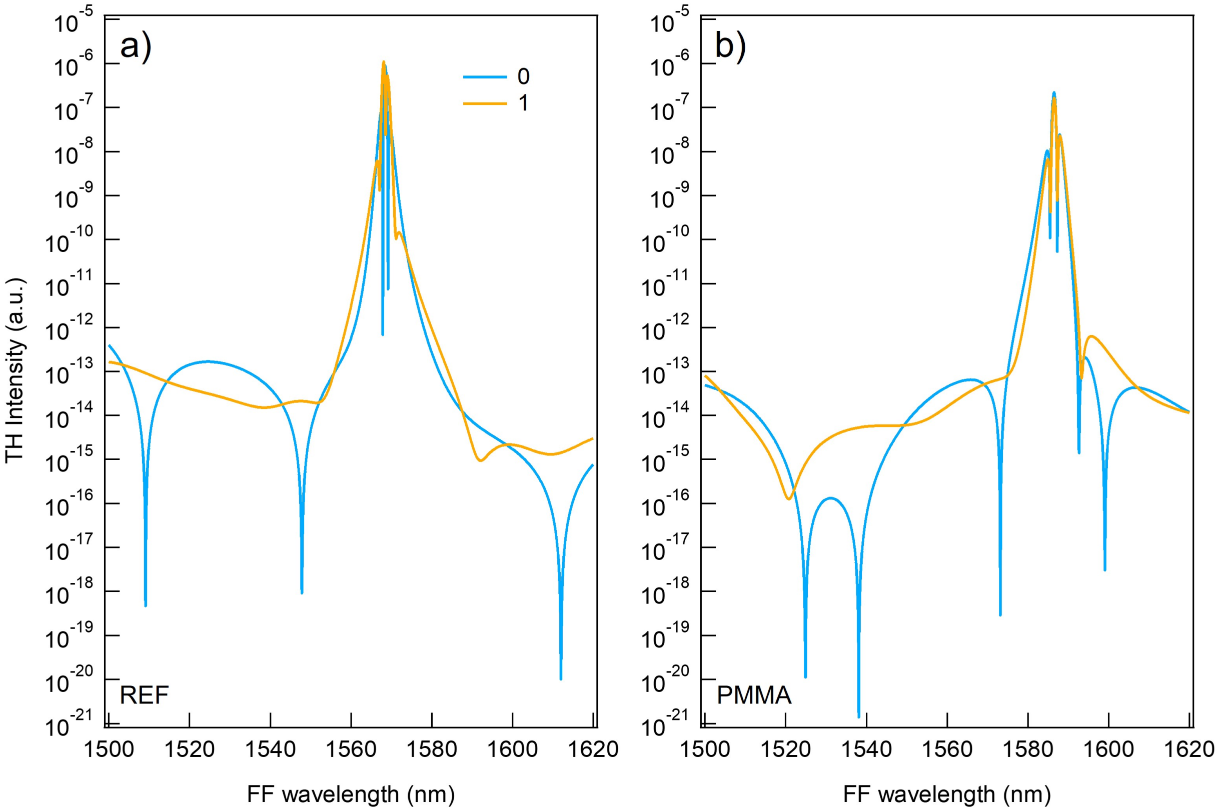

FIGURE S10. **TH Emission through Diffraction Orders.** Calculated intensity of the TH radiation emitted trough diffraction channels of order 0 (light blue solid line) and +1 (yellow solid line) as a function of the FF excitation wavelength for the REF (panel a) and PMMA (panel b) sample.

In order to better reproduce the experimental condition, the values of the ratio $I^{(1)}/I^{(0)}$ displayed in Figure 5g and h of the main text (markers and dashed line) have been calculated taking into account the fact that the FF excitation has a finite bandwidth. In particular, starting from the numerical data in Fig. S10 (*i.e.*, $I_{\mathrm{TH}}^{(m)}(\lambda)$ with $m = 0, 1$), we calculated

$$\frac{I^{(1)}(\lambda_{\mathrm{FF}})}{I^{(0)}(\lambda_{\mathrm{FF}})} = \frac{\int_{-\infty}^{+\infty} \mathrm{d}\lambda\, \mathcal{G}(\lambda; \lambda_{\mathrm{FF}})\ I_{\mathrm{TH}}^{(1)}(\lambda)}{\int_{-\infty}^{+\infty} \mathrm{d}\lambda\, \mathcal{G}(\lambda; \lambda_{\mathrm{FF}})\ I_{\mathrm{TH}}^{(0)}(\lambda)}, \tag{S10}$$

where the distribution function $\mathcal{G}(\lambda; \lambda_{FF})$ consists in a normalized Gaussian profile of the form

$$\mathcal{G}(\lambda; \lambda_{\mathrm{FF}}) = \sqrt{\frac{4 \cdot \log 2}{\pi \Delta\lambda_{\mathrm{TH}}^2}} \cdot \exp\left[-\frac{4 \cdot \log 2}{\Delta\lambda_{\mathrm{TH}}^2} \cdot (\lambda_{\mathrm{FF}} - \lambda)^2\right]$$

with $\Delta\lambda_{TH}$ being the full width at half maximum of the spectral intensity profile of the TH radiation resulting from the excitation with a FF radiation with a spectrum centered at λ_{FF} with bandwidth $\Delta\lambda_{FF}$. The choice of $\Delta\lambda_{TH}$ as the bandwidth of the distribution function takes into account the third-order nature of the process under investigation and its value has been set according to Sec. II D.

[1] R. W. Boyd, *Nonlinear Optics* (Academic Press, 2003).

[2] U. Fano, Phys. Rev. **124**, 1866 (1961).
